# Linking early and late endpoints in adjuvant immunotherapy trials: an evaluation of multiple clinical outcomes

**DOI:** 10.1093/oncolo/oyag342

**Published:** 2026-08-22

**Authors:** Sirui Tang, Huiran Zang, Linhan Mao, Xingyu Liu, Xinyu Li

**Affiliations:** Department of Plastic Surgery, Peking University People’s Hospital, No. 11 Xizhimen South Street, Xicheng, Beijing, 100044, China; Department of Plastic Surgery, Peking University People’s Hospital, No. 11 Xizhimen South Street, Xicheng, Beijing, 100044, China; Yunnan Normal University, Kunming, Yunnan, China; University of Leeds, Leeds, United Kingdom; Yunnan Normal University, Kunming, Yunnan, China

**Keywords:** adjuvant immunotherapy, disease-free survival, recurrence-free survival, progression-free survival, overall survival, quality of life

## Abstract

**Background:**

Overall survival (OS) in adjuvant and perioperative immunotherapy trials requires prolonged follow-up. We examined whether treatment effects on disease-free survival (DFS) and recurrence-free survival (RFS) were associated with OS at matched 2-, 3-, and 5-year windows.

**Methods:**

The main analysis included 23 strict-primary randomized trials. DFS and RFS were modeled separately across 6 paired time windows using 2 co-primary trial-level approaches: a conventional OS-variance-weighted regression and a conditional errors-in-variables model accounting for uncertainty in both log-HRs. Because within-trial correlations were unavailable, ρ was varied from 0 to 0.75. Eligibility-sensitivity studies were supplementary; broad-taxonomy evidence was excluded. EFS was descriptive, PFS and PFS2 were analyzed separately, and QoL regression required at least 5 verified contrasts.

**Results:**

Nineteen trials contributed 2-year same-window pairs (12 DFS; 7 RFS), 8 contributed 3-year pairs, and 5 contributed 5-year pairs. For 2-year early endpoints vs 2-year OS, weighted slopes were 0.014 (95% CI, −0.286 to 0.314) for DFS and 0.630 (95% CI, −1.243 to 2.502) for RFS. Eight of 9 fitted weighted-regression intervals included zero; the only nominally positive result involved 4 RFS trials. In errors-in-variables analyses, slopes for the largest DFS and RFS comparisons ranged from 0.011 to 0.264 and from 0.758 to 0.885, respectively, and all profile CIs included zero. Six strict-primary QoL contrasts were verified, but no window reached *n* = 5.

**Conclusion:**

Strict-primary analyses did not demonstrate a consistent or precise early-endpoint–OS relationship. Sparse later windows and uncertainty in both treatment-effect estimates preclude surrogate validation.

Implications for practice The main analysis was restricted to 23 trials meeting the prespecified eligibility criteria and modeled disease-free survival and recurrence-free survival separately. The conventional overall survival (OS)-variance-weighted regression was retained for comparability, while the co-primary conditional errors-in-variables analysis was the principal model addressing uncertainty in both hazard ratio estimates. Neither supported a stable treatment-effect relationship. Strict-primary quality of life evidence was too sparse for regression. These aggregate findings do not validate an early endpoint as a surrogate for OS.

## Introduction

Adjuvant immunotherapy has increasingly shifted curative-intent treatment for solid tumors toward perioperative and postoperative strategies.^1^^,^^2^ Its use has expanded across melanoma, lung cancer, esophageal cancer, and several other tumor types, reflecting consistent improvements in recurrence outcomes.^3^ Despite these advances, overall survival (OS) remains the gold standard endpoint but is difficult to assess in modern adjuvant trials.^4^ Long survival after recurrence, access to effective salvage immunotherapy, and the need for extended follow-up all limit the feasibility of OS as a primary endpoint in this setting.

Earlier endpoints such as recurrence-free survival (RFS), disease-free survival (DFS), and event-free survival (EFS) enable more timely evaluation of adjuvant therapies, whereas progression-free survival (PFS) is used more often in settings with measurable or residual disease. Their event definitions are not identical, and an early endpoint may be clinically meaningful without predicting the magnitude of the OS treatment effect.^5^^,^^6^ Evaluation across explicitly matched follow-up windows can show whether the aggregate treatment–effect relationship changes as OS matures.

Quality of life (QoL) is an important component of benefit in early-stage cancer, particularly when patients are disease free after definitive therapy. However, QoL publications vary in domain, assessment time, estimand, completion, and missing-data handling.^7–9^ Global health/QoL contrasts therefore require consistent direction and should be related separately to early-endpoint and OS effects.

The primary objective was to evaluate aggregate associations between DFS/RFS and OS treatment effects in adjuvant and perioperative immunotherapy trials across matched 2-, 3-, and 5-year windows, including robustness to uncertainty in both endpoint estimates. Secondary objectives were to describe EFS, PFS, PFS2, and global health/QoL within analyses supported by available data. The study was not intended to establish individual-level surrogacy or validate a universal early endpoint for OS.

## Methods

The INPLASY record (INPLASY2025110071; doi: 10.37766/inplasy2025.11.0071), registered on November 21, 2025 after review completion but before publication, is retrospective. Protocol-to-manuscript differences are detailed in the Supplementary Tables.

### Search strategy and selection criteria

PubMed, Embase, Web of Science, and the Cochrane Library were searched for English-language reports. The protocol specified peer-reviewed Phase II or III randomized controlled trials published from 2016 onward in adults with resectable solid tumors treated with adjuvant or perioperative immunotherapy after curative-intent therapy. Eligible comparators were placebo, observation, or postoperative management without adjuvant immunotherapy. Complete search strategies are provided in Table S1, and all 44 submitted source records and dispositions are documented in Table S2.

### Data extraction and outcomes

Two reviewers independently extracted trial registration, randomized comparison, endpoint definition, analysis population, data cutoff, follow-up, treatment direction, HR, and 95% CI. The source hierarchy was prespecified: exact values in the primary or updated publication were preferred, followed by exact supplementary tables, main or supplementary figure annotations, and curve-based reconstruction only when no usable time-specific HR was reported. Dedicated QoL publications were linked to the same randomized comparison. Disagreements in trial identity, arm direction, cutoff, endpoint family, or source location were resolved by consensus before analysis.

For curve-based extraction, the relevant Kaplan–Meier panel and its numbers-at-risk table were reviewed at the highest available resolution. The WebPlotDigitizer web application (Automeris; https://automeris.io/WebPlotDigitizer) was used to calibrate the horizontal time axis and vertical survival-probability axis from published tick marks. Treatment and control curves were traced separately. Short censoring marks and non-curve graphical elements were excluded from the trace. Digitized landmark probabilities were cross-checked against printed survival estimates and the monotonic sequence of numbers at risk. A curve was not used when the panel, arm identity, axis calibration, or risk table could not be verified.

When the required HR was unavailable, digitized arm-specific survival probabilities at the target window were used as intermediate inputs to reconstruct an approximate cumulative-hazard ratio and 95% CI with the published numbers at risk, following the Parmar framework.^10^ Reconstruction did not create pseudo-individual-patient data and was not used when an exact same-population HR was available. Every effect estimate was labeled as directly reported or reconstructed. Early-endpoint and OS effects were paired only when trial, randomized contrast, arm direction, analysis population, and time window agreed. Final regressions used HRs with 95% CIs rather than arm-level survival percentages.

DFS, RFS, EFS, and PFS retained the labels and event definitions used by the investigators. PFS and PFS2 were recorded separately from recurrence endpoints. QoL extraction was restricted to EORTC QLQ-C30 global health/QoL on its 0-100 scale. Contrasts were recoded as treatment minus control, with higher values indicating better global health. Functional and symptom scales were not combined with global health/QoL. The assessment window, longitudinal estimand, analysis population, completion, missing-data handling, CI, and exact source were recorded for each contrast.

### Risk of bias assessment

Risk of bias was assessed independently by 2 reviewers using Cochrane RoB 2.^11^ Judgments were made separately for the recurrence endpoint, OS, and QoL. For QoL, missing patient-reported outcomes, postbaseline availability, analysis population, and selective reporting of domains or time points were considered explicitly. Outcome-specific domain judgments and rationales are reported in Table S3.

### Statistical analysis

#### Endpoint-specific aggregate association analysis

The main analysis was restricted to strict-primary trials. DFS and RFS were modeled separately, retaining each trial’s reported endpoint label and definition. EFS was not regressed because no strict-primary trial contributed a valid EFS–OS pair. Only same-trial, same-contrast, same-population early-endpoint and OS effects were paired. These aggregate models do not evaluate individual-level surrogacy.^12^^,^^13^

The 2-year cohort contained every strict-primary trial eligible for a 2-year comparison; the 3-year cohort was a subset of the 2-year cohort, and the 5-year cohort was a subset of both. OS was death from any cause. Trials without a usable pair remained in the trial evidence set but did not contribute to that time-window regression.

Analyses were performed using R version 4.5.2 with metafor and base statistical functions.

#### Co-primary trial-level association models

For each endpoint and window, x = −log(HR) for DFS or RFS and y = −log(HR) for OS, so positive values favored immunotherapy. The conventional OS-variance-weighted regression was retained as a co-primary comparability analysis. The conditional errors-in-variables analysis was the principal co-primary model addressing uncertainty in both estimates and their assumed within-trial correlation; this model and the ρ grid were added after peer review and were not specified in the INPLASY record.

Six comparisons were prespecified for each endpoint family: 2-year early endpoint vs 2-, 3-, and 5-year OS; 3-year early endpoint vs 3- and 5-year OS; and 5-year early endpoint vs 5-year OS. Models required at least 3 usable pairs and predictor variation. Weighted-regression slope CIs, unadjusted 2-sided *P* values, weighted *R*², adjusted *R*², cross-validated *R*², and 1000-resample trial bootstrap results were reported for comparability; *n* = 3-4 models were explicitly exploratory.

For trial i, the treatment effects on the early endpoint and OS were expressed as


$$x_{i}=-\log({\mathrm{HR}}_{\mathrm{early},i}),\;y_{i}=-\log({\mathrm{HR}}_{\mathrm{OS},i}).$$

Let sxi and syi denote the corresponding standard errors. For each fixed within-trial correlation ρ ∈ {0, 0.25, 0.50, 0.75}, a conditional errors-in-variables model was specified as


$$\begin{array}{l} y_{i}=\alpha +\beta x_{i}+\varepsilon_{i},\;\varepsilon_{i}∼N(0,V_{i}).\\ V_{i}={s_{y_{i}}}^{2}+\beta^{2}{s_{x_{i}}}^{2}-2\beta \rho s_{x_{i}}s_{y_{i}}+\tau^{2}. \end{array}$$

For a fixed value of ρ, the model parameters α, β, and τ were estimated by minimizing the negative log-likelihood


$$\mathrm{NLL}(\alpha ,\beta ,\tau )=\frac{1}{2}\sum_{i=1}^{n}\left[\log(2\pi V_{i})+\frac{{\left(y_{i}-\alpha -\beta x_{i}\right)}^{2}}{V_{i}}\right].$$

A custom base-R routine jointly estimated α, β, and log(τ) by maximum likelihood using optim(method = ‘L-BFGS-B’). Profile-likelihood 95% CIs for β were obtained by fixing β, reoptimizing α and log(τ), and solving the likelihood-ratio boundary


$$2[{\mathrm{NLL}}_{p}(\beta )-{\mathrm{NLL}}_{\min}]=\chi_{1,0.95}^{2},$$

using uniroot. A confidence limit was reported as not estimable when profiling failed or no cutoff crossing was found within the prespecified β search range; no value was imputed. The null hypothesis H0: β = 0 was evaluated using the likelihood-ratio statistic


$$\Lambda =2[{\mathrm{NLL}}_{\beta =0}-{\mathrm{NLL}}_{\min}]∼\chi_{1}^{2}.$$

In addition, uncertainty was assessed using 1000 trial-level bootstrap resamples.

Additional sensitivities added only eligibility-sensitivity studies, excluded every pair containing a reconstructed HR, and retained the originally specified pooled DFS/RFS regression for comparison. Broad-taxonomy evidence was excluded from all quantitative models.

EFS was summarized descriptively. PFS and PFS2 were recorded separately; a PFS-only supplementary analysis excluded the single PFS2 study, and the mixed PFS/PFS2 model was retained only as a legacy supplementary sensitivity. No strict-primary PFS regression was fitted because fewer than 3 strict pairs were available.

#### QoL evidence and exploratory meta-regression

The primary QoL evidence was restricted to source-verified exact EORTC QLQ-C30 global-health/QoL contrasts from strict-primary trials. Contrasts were coded as treatment minus control, with higher values indicating better global health. A REML meta-regression was fitted only when at least 5 verified contrasts were available for the time window. For *n* < 5, only the contrast count and trial-level estimates were reported; slopes, *P* values, *R*², and leave-one-out refits were not calculated. Verified contrasts from eligibility–sensitivity studies were confined to a supplementary sensitivity, and 4 provisional contrasts were descriptive only.

#### Subgroup analysis

The originally planned subgroup analyses were not used for inferential conclusions after endpoint harmonization because most strata contained too few trials for reliable interaction testing. Trial phase, disease setting, treatment class, and follow-up were retained as descriptive characteristics in Table S2.

### Additional sensitivity analyses

Additional sensitivity analyses comprised leave-one-out refitting when at least 4 trials were available, eligibility-expanded endpoint-specific models, and a direct-HR-only analysis requiring both HRs in a pair to have been directly reported. Because endpoint-specific direct pairs were sparse, DFS and RFS were pooled only for that explicitly labeled source-restriction sensitivity.

Eligibility–sensitivity studies appeared only in supplementary analyses. The broad-taxonomy trial was retained in the audit ledger but excluded from all quantitative datasets. Results were interpreted from effect magnitude, uncertainty, cohort size, endpoint definition, source type, and predictive performance rather than from *R*² alone.

## Results

### Study characteristics and risk of bias

**Figure 1.**
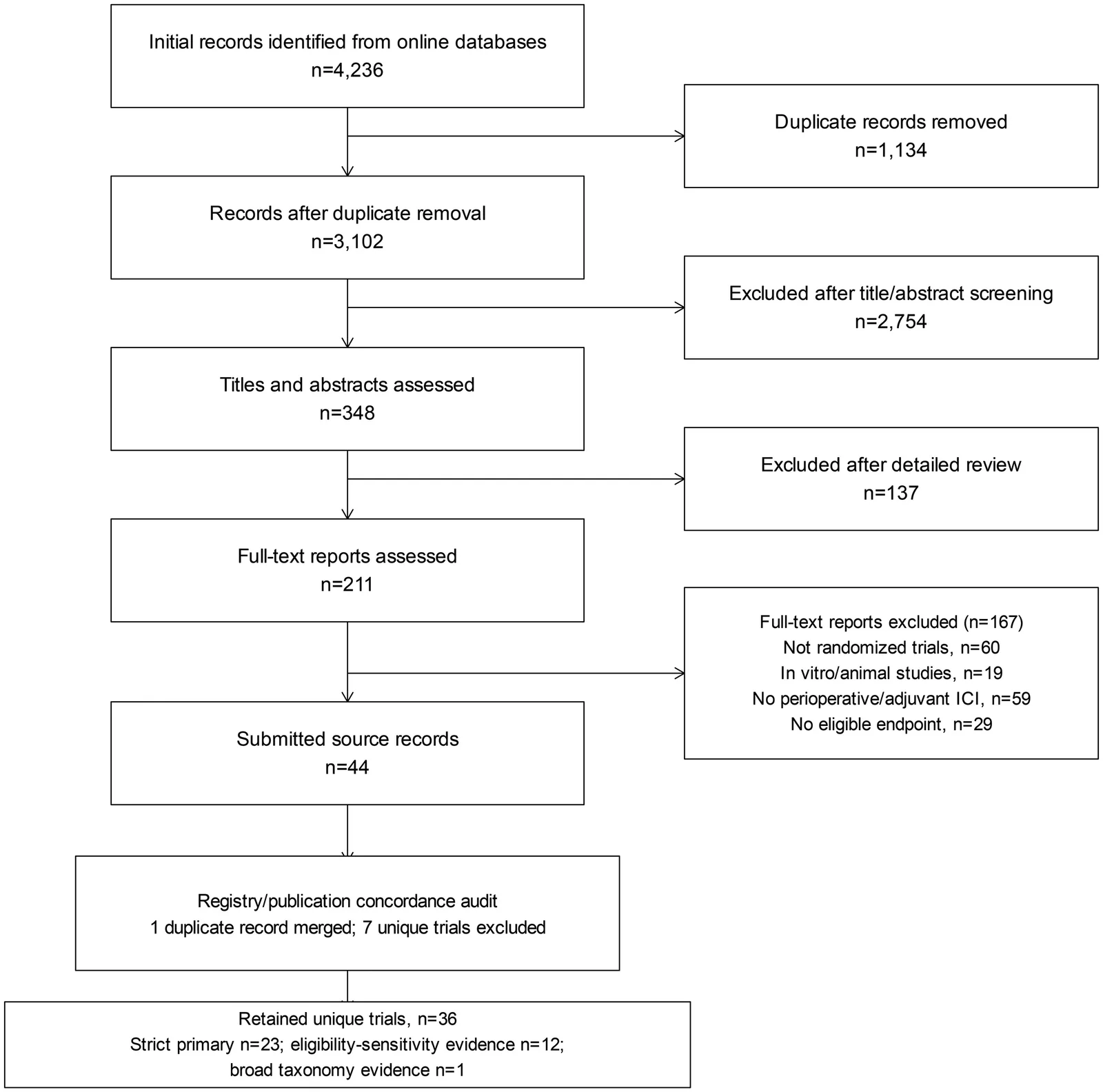
Study-selection flow and registry/publication concordance audit. The original search identified 4236 records and yielded 44 submitted source records. The audit merged one duplicate record and excluded 7 unique trials, leaving 23 strict-primary trials for the main quantitative analysis, 12 eligibility-sensitivity trials for supplementary analyses, and one broad-taxonomy trial retained only in the descriptive audit.

The 44 submitted entries represented source records rather than independent trials. The audit resolved duplicate, subgroup-only, registration-mismatched, and ineligible records. One duplicate was merged and 7 unique trials were excluded. The 23 strict-primary trials^14–36^ defined the main analysis; 12 eligibility-sensitivity trials were reserved for supplementary analyses, and one broad-taxonomy trial was descriptive only^37–49^ (Figure 1; Table S2).

The strict-primary set comprised 4 phase II and 19 phase III trials with 17,112 randomized participants. Disease composition is shown in Figure S1, and endpoint family, OS status, paired-analysis contribution, verified QoL availability, and randomized sample size are shown in Figure S2.

Nineteen strict-primary trials contributed a 2-year same-window pair: 12 DFS and 7 RFS. Eight contributed a 3-year same-window pair: 4 DFS and 4 RFS. Five contributed a 5-year same-window pair: 3 DFS and 2 RFS. No strict-primary EFS trial contributed a valid pair. In the complete extraction ledger, 112 time-specific endpoint cells comprised 66 directly reported and 46 Kaplan–Meier reconstructed HRs. The source status of every cell was retained for direct-HR-only sensitivity analysis (Figure S2; Table S2).

For recurrence endpoints, the outcome-specific RoB 2 panel showed low risk or some concerns across all domains. The OS panel showed the same 2 judgment categories. QoL assessments had some concerns for missing outcome data, outcome measurement, selection of the reported result, and overall bias (Figure S3; Table S3).

### Time-specific associations of DFS and RFS with OS

**Figure 2.**
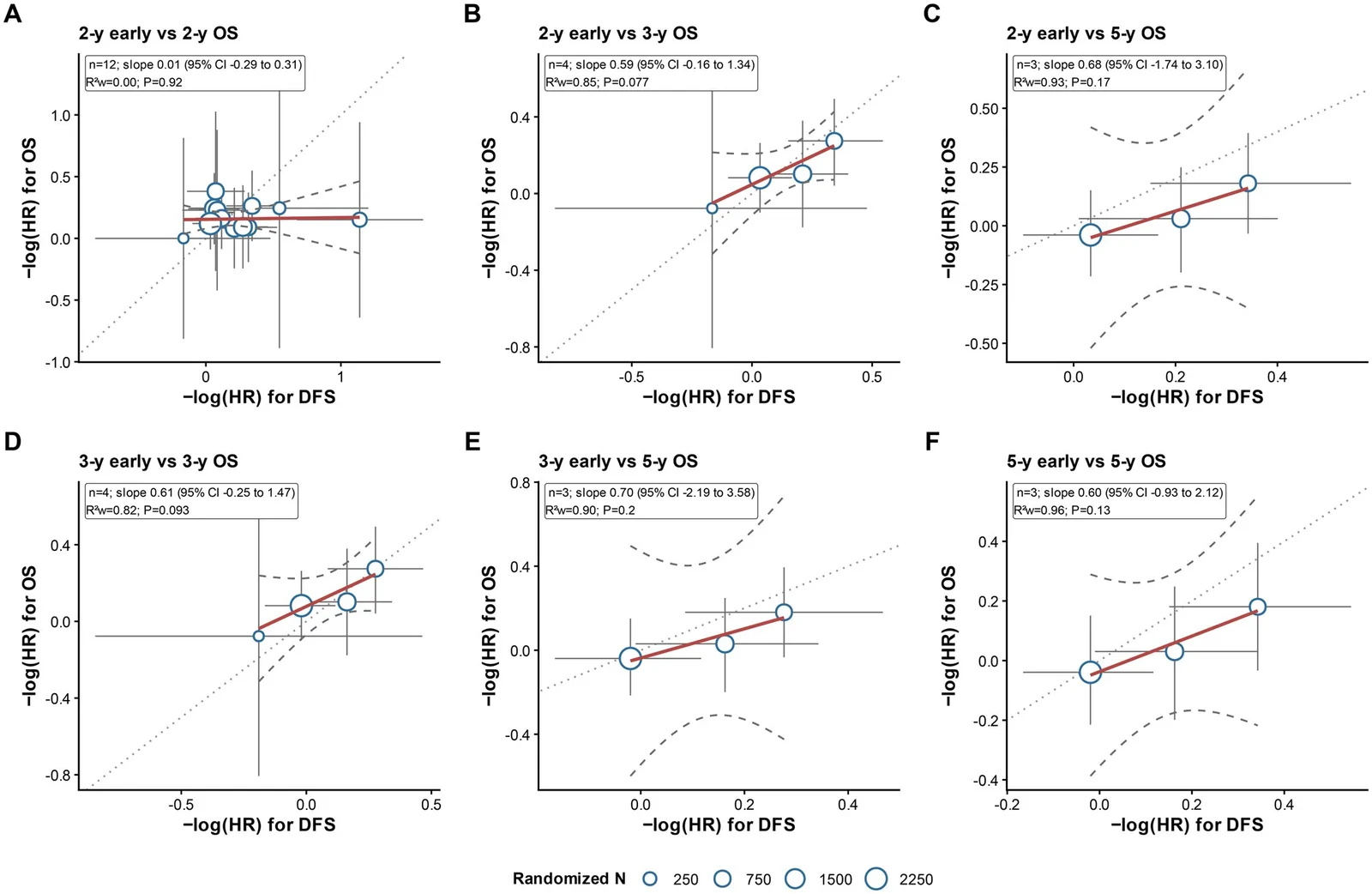
Strict-primary DFS–OS associations at 6 prespecified time-window comparisons: (A) 2-year DFS vs 2-year OS; (B) 2-year DFS vs 3-year OS; (C) 2-year DFS vs 5-year OS; (D) 3-year DFS vs 3-year OS; (E) 3-year DFS vs 5-year OS; and (F) 5-year DFS vs 5-year OS. Horizontal and vertical bars show 95% CIs. Red lines show inverse-OS-variance-weighted regressions, gray dashed lines show model-based 95% confidence limits, and gray dotted lines indicate equality. Circle area is proportional to randomized sample size.

In the conventional OS-variance-weighted comparability analysis, the strict-primary 2-year DFS–2-year OS model included 12 trials and gave slope 0.014 (95% CI −0.286 to 0.314), weighted *R*² = 0.001, and *P* = .918 (Figure 2A). The remaining DFS slopes ranged from 0.591 to 0.695; all 5 model-based 95% CIs included zero, with only 3 or 4 trials in each later-window comparison (Figure 2B–F; Table 1).

**Table 1.** Strict-primary endpoint-specific OS-variance-weighted comparability analyses between time-specific early endpoints and overall survival.

Endpoint	Comparison	Trials, *n*	Slope (model-based 95% CI)	*P* value	Weighted *R*² (bootstrap 95% CI)	Cross-validated *R*²	Interpretation
**DFS**	2y early vs 2 y OS	12	0.014 (−0.286 to 0.314)	.918	0.001 (0-0.361)	−0.418	95% CI includes zero.
**DFS**	2y early vs 3 y OS	4	0.591 (−0.161 to 1.344)	.077	0.851 (0.492-1)	−0.045	Exploratory only because *n* < 5; nominal *P* value without multiplicity adjustment; not evidence of surrogacy.
**DFS**	2y early vs 5 y OS	3	0.681 (−1.737 to 3.098)	.174	0.927 (0.927-1)	—	Exploratory only because *n* < 5; nominal *P* value without multiplicity adjustment; not evidence of surrogacy.
**DFS**	3y early vs 3 y OS	4	0.608 (−0.25 to 1.465)	.093	0.823 (0.43-1)	−0.269	Exploratory only because *n* < 5; nominal *P* value without multiplicity adjustment; not evidence of surrogacy.
**DFS**	3y early vs 5 y OS	3	0.695 (−2.194 to 3.584)	.201	0.903 (0.903-1)	—	Exploratory only because *n* < 5; nominal *P* value without multiplicity adjustment; not evidence of surrogacy.
**DFS**	5y early vs 5 y OS	3	0.596 (−0.928 to 2.121)	.126	0.961 (0.961-1)	—	Exploratory only because *n* < 5; nominal *P* value without multiplicity adjustment; not evidence of surrogacy.
**RFS**	2y early vs 2 y OS	7	0.63 (−1.243 to 2.502)	.427	0.13 (0-0.891)	−0.38	95% CI includes zero.
**RFS**	2y early vs 3 y OS	4	1.011 (0.416 to 1.607)	.018	0.964 (0.947-1)	0.881	Exploratory only because *n* < 5; nominal *P* value without multiplicity adjustment; not evidence of surrogacy.
**RFS**	2y early vs 5 y OS	2	Not fitted	—	—	—	Not fitted (*n* < 3).
**RFS**	3y early vs 3 y OS	4	0.66 (−0.077 to 1.398)	.061	0.881 (0.873-1)	0.436	Exploratory only because *n* < 5; nominal *P* value without multiplicity adjustment; not evidence of surrogacy.
**RFS**	3y early vs 5 y OS	2	Not fitted	—	—	—	Not fitted (*n* < 3).
**RFS**	5y early vs 5 y OS	2	Not fitted	—	—	—	Not fitted (*n* < 3).

The main analysis is restricted to the 23 strict-primary trials and models DFS and RFS separately. This table presents the conventional OS-variance-weighted regression retained as a co-primary comparability analysis. Conditional errors-in-variables co-primary results are reported in Table S5 and Figure S7. *P* values are unadjusted across the 9 fitted endpoint/window models. All *n* = 3-4 results are exploratory; a high *R*² or nominal *P* value in such cohorts is not evidence of surrogacy. No strict-primary EFS regression was fitted.

**Figure 3.**
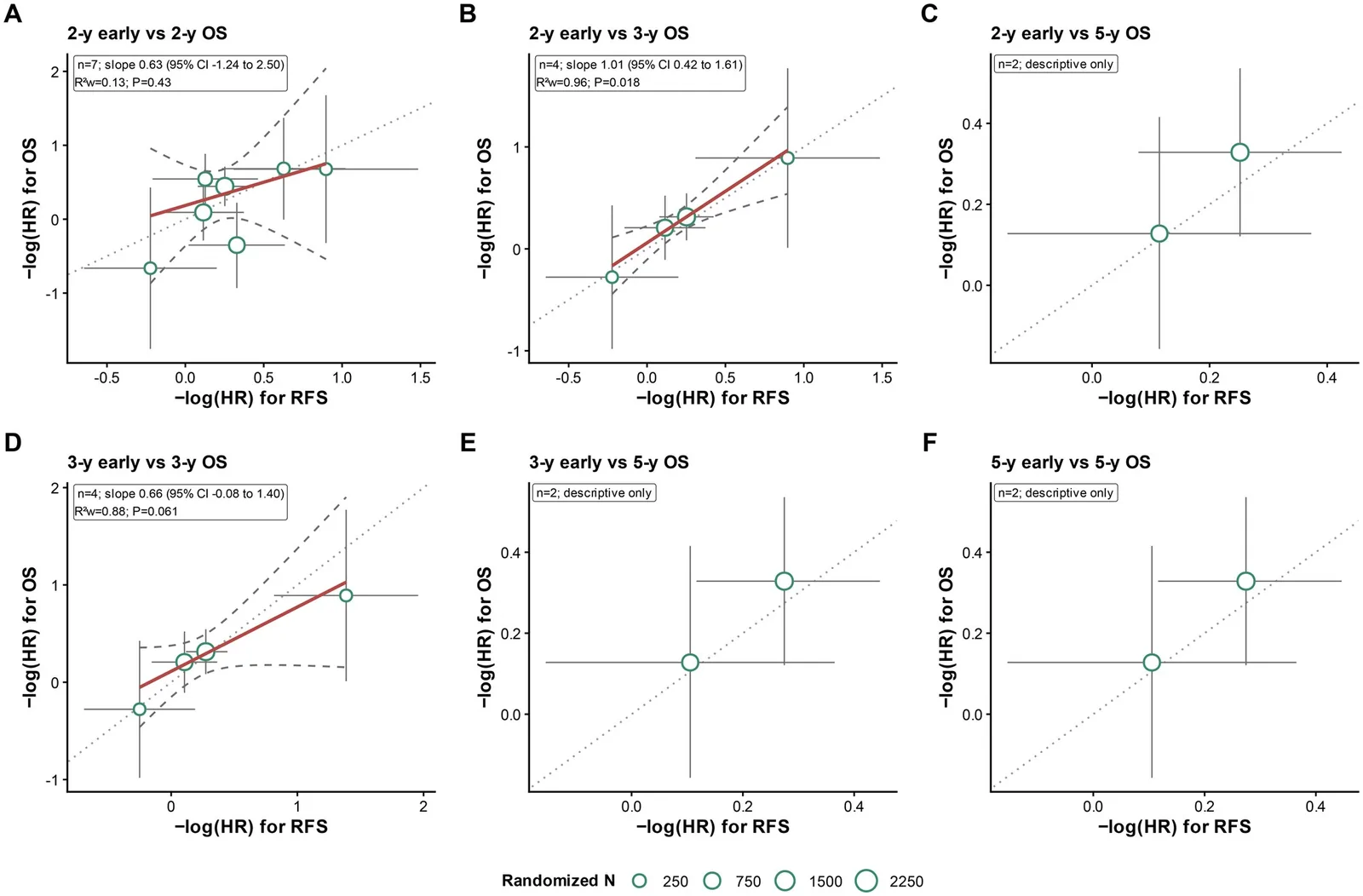
Strict-primary RFS–OS associations at the same 6 prespecified time-window comparisons and in the same panel order as Figure 2. Regressions are shown only when at least 3 usable pairs were available. Panels C, E, and F each contain 2 trials and are descriptive only. Horizontal and vertical bars show 95% CIs; red lines show inverse-OS-variance-weighted regressions; gray dashed lines show model-based 95% confidence limits; and gray dotted lines indicate equality.

In the weighted RFS comparability analysis, the 2-year RFS–2-year OS model included 7 trials and gave slope 0.630 (95% CI −1.243 to 2.502), weighted *R*² = 0.130, and *P* = .427 (Figure 3A). The 4-trial 2-year RFS–3-year OS model gave slope 1.011 (95% CI 0.416-1.607; unadjusted nominal *P* = .018), whereas the 4-trial 3-year RFS–3-year OS interval included zero (slope 0.660, 95% CI −0.077 to 1.398; *P* = .061; Figure 3B, D). Both were exploratory because *n* < 5. The other 3 RFS comparisons each contained 2 trials and were descriptive only (Figure 3C, E, F; Table 1).

Thus, 8 of 9 fitted intervals in the weighted comparability analysis included zero. The single nominally positive result arose from 4 RFS trials, was unadjusted for multiplicity, and was not treated as confirmatory or as evidence of surrogacy. Trial-specific same-window HRs are shown in Figure S4. Bootstrap distributions for the 9 fitted strict-primary models are shown in Figure S5.

EFS was not regressed in the main analysis because no strict-primary EFS pair was available.

### Descriptive QoL evidence and availability for survival comparisons

**Figure 4.**
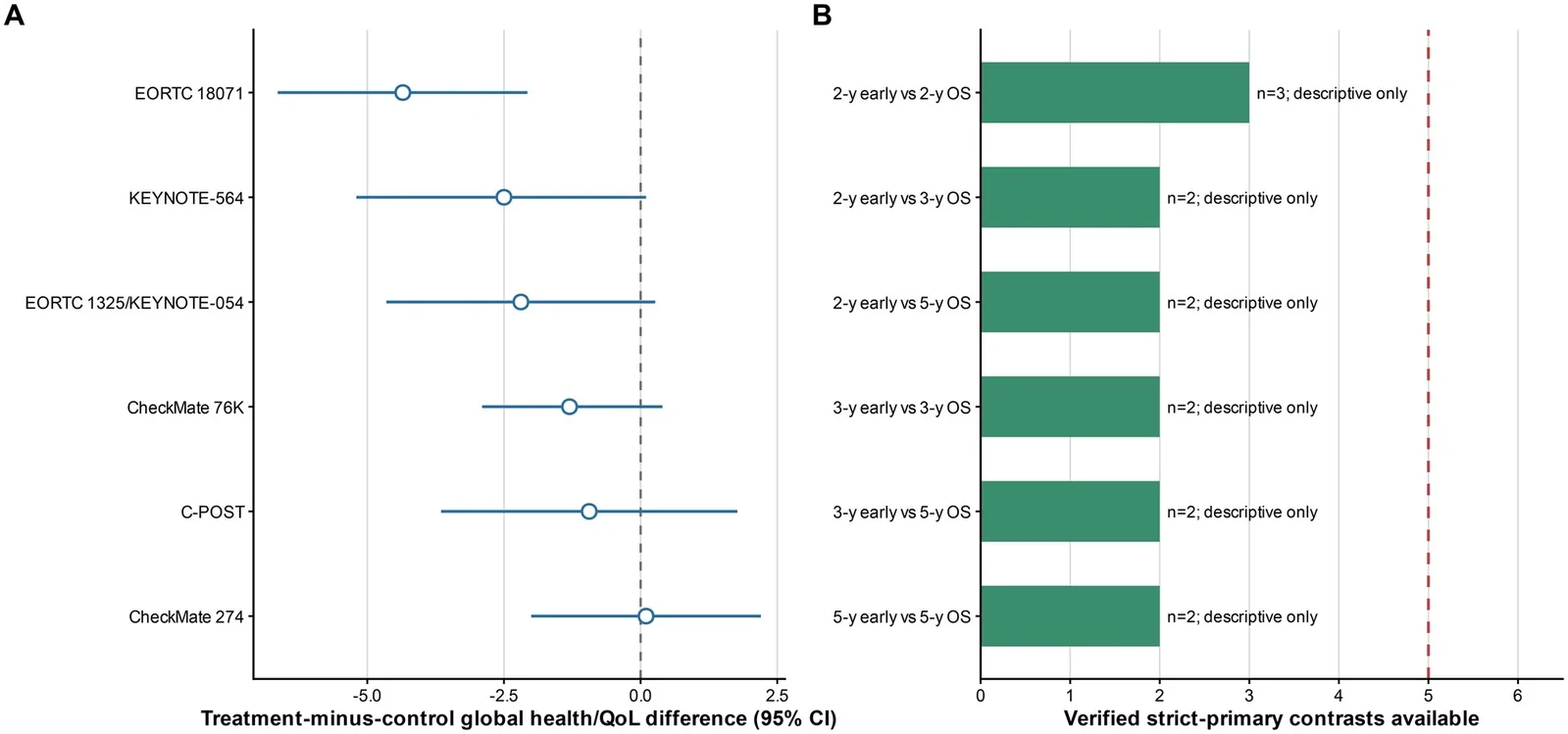
Strict-primary verified global-health/QoL evidence. (A) Six source-verified treatment-minus-control global-health/QoL mean differences with 95% CIs; higher values favor better global health/QoL. (B) Number of verified strict-primary contrasts available for each survival comparison. The dashed red line marks the prespecified minimum of 5 contrasts for regression. Every comparison remained below this threshold, so no primary QoL slope, *P* value, or *R*² was estimated.

Six strict-primary trials had source-verified exact global-health/QoL contrasts (Figure 4A; Table S4). Only 3 contributed to the 2-year same-window survival pairing, and every other time comparison had two. Because no comparison reached the prespecified minimum of 5 contrasts (Figure 4B), no primary QoL regression slope, *P* value, or *R*² was estimated.

Two additional verified exact contrasts from eligibility–sensitivity trials and 4 provisional contrasts were retained only in the supplementary material. Provisional values did not enter any regression (Figure S6A–D; Table S4).

### Strict-primary PFS/PFS2 evidence

Only ACT IV contributed a strict-primary conventional PFS–OS pair, whereas CheckMate 274 contributed a strict-primary PFS2–OS pair. Because PFS and PFS2 represent different estimands and the strict-primary conventional PFS sample contained only one trial, no strict-primary regression was fitted (Figure S6F; Table S5).

After eligibility-sensitivity studies were added, the PFS-only model excluded CheckMate 274 PFS2 and contained 4 trials; its slope was 0.575 (95% CI −0.290 to 1.440), weighted *R*² = 0.804, and *P* = .103 (Figure S6E; Table S5). The legacy 5-trial PFS/PFS2 model gave slope 0.639 (95% CI 0.145-1.134), weighted *R*² = 0.850, and *P* = .026, but remained a supplementary mixed-estimand analysis rather than evidence from the prespecified strict-primary population.

### Co-primary measurement-error and additional sensitivity analyses

Trial-bootstrap distributions for the weighted comparability analysis were broad or multimodal in several 3- and 4-trial models (Figure S5). In the co-primary conditional errors-in-variables analysis, the 12-trial DFS same-window slope ranged from 0.011 at ρ = 0 to 0.264 at ρ = 0.75, and every profile 95% CI included zero. The 7-trial RFS same-window slope ranged from 0.758 to 0.885, again with every profile CI including zero. Some *n* = 3-4 fits excluded zero only under particular ρ assumptions, while other profile bounds were not estimable; these unadjusted, assumption-specific findings were exploratory (Figure S7; Table S5).

The strict direct-HR-only pooled models contained only 3 or 4 trials in each estimable comparison, and every model-based slope CI included zero. Eligibility-expanded DFS/RFS models, direct-HR-only models, and the 2 EFS trials are reported in Figure S8 and Table S5; the broad-taxonomy trial was excluded throughout.

Adding the 2 eligibility-sensitivity verified QoL contrasts produced the only estimable QoL sensitivities (*n* = 5). The slopes against the 2-year early-endpoint and 2-year OS effects were −1.304 (95% CI −15.273 to 12.664; *P* = .855) and −1.030 (95% CI −38.126 to 36.067; *P* = .957), respectively, with no evidence of a stable association (Figure S6C, D; Tables S4–S5). The 4 provisional QoL contrasts remained descriptive only (Figure S6B).

## Discussion

The principal finding is that neither co-primary analysis demonstrated a stable relationship between early-endpoint and OS treatment effects. The conventional OS-variance-weighted regression was retained for comparability with the original analysis, whereas the conditional errors-in-variables model was the principal analysis addressing uncertainty in both HR estimates and their assumed within-trial correlation. A convincing relationship would require consistent slopes, informative intervals, out-of-sample performance, and stability across definitions and windows. Instead, most intervals included zero and the only nominal weighted-regression result came from 4 RFS trials. The evidence supports uncertainty rather than a quantitative conversion from an early-endpoint HR to an OS HR.

The pattern across windows is informative. The 12-trial DFS same-window model was nearly null, whereas stronger later-window DFS slopes used only 3 or 4 trials. The 7-trial RFS same-window estimate was imprecise, and the nominal 4-trial RFS result was not reproduced across other windows. Favorable later slopes therefore coincided with smaller cohorts and greater leverage, not progressively clearer alignment as OS matured. High weighted R² values in these sparse models describe the fitted sample, not transportable prediction.

Endpoint separation is clinically important because DFS, RFS, EFS, PFS, and PFS2 are different estimands. DFS may count recurrence, second cancers, or death; RFS definitions vary in handling death and new primaries; and PFS2 concerns progression after subsequent therapy. These differences alter event frequency and causal pathways to OS. DFS validation in adjuvant colon cancer arose within a more homogeneous framework.^50^ Melanoma analyses also show that patient-level association may exceed trial-level treatment–effect association and have not established a universal checkpoint-inhibitor RFS surrogate.^51^^,^^52^

The cross-disease design further limits transportability. Baseline prognosis, recurrence pattern, salvage options, competing mortality, and OS maturity differ across tumors. Pooling settings can increase sample size while obscuring effect modification. Endpoint-specific analyses reduce one source of heterogeneity but cannot make a common slope exchangeable across indications. The results therefore do not show that recurrence endpoints lack value; they show that a cross-tumor mapping to OS is unsupported.

The supplementary PFS findings illustrate this distinction. Conventional PFS and PFS2 represent different estimands and should not be pooled without qualification. Evidence that PFS can track OS in advanced non-small-cell lung cancer does not validate a mixed adjuvant and residual-disease construct.^53^ In this review, only one strict-primary conventional PFS pair was available. The 4-trial PFS-only sensitivity was imprecise, whereas the nominally positive 5-trial legacy result appeared only after PFS2 was combined with PFS and eligibility was expanded. Its statistical significance therefore does not overcome the mismatch in estimands or establish applicability to the prespecified population.

Association is not surrogacy. Validation requires credible patient-level association, a strong randomized treatment–effect relationship, calibration for a new trial, and transportability to the intended setting.^5^^,^^54^ Aggregate published data cannot estimate patient-level association, separate trial error from heterogeneity precisely, define a reliable surrogate threshold effect, or test external prediction. A nominal *P* value or high in-sample *R*² from 3 or 4 trials meets none of these requirements and may change when one trial is removed.

Several clinical pathways can decouple recurrence control from death. Effective salvage therapy, surgery for isolated recurrence, treatment crossover, competing mortality, and long post-recurrence survival can attenuate or delay an OS contrast even when recurrence is reduced. Conversely, toxicity-related mortality or differences in post-progression care could affect OS without a proportional change in recurrence. In modern melanoma, the evolving post-recurrence treatment landscape is one reason adjuvant benefit cannot be summarized by a fixed RFS-to-OS conversion.^55^ Recurrence and OS should therefore be treated as related but distinct outcomes.

Follow-up duration is part of the estimand. Longer observation allows more deaths to accrue but also changes post-recurrence management, proportional-hazards behavior, and the trials eligible for analysis. Earlier colon-cancer work found 2- or 3-year DFS informative for later OS under tightly constrained definitions.^56^ Here, matching 2-, 3-, and 5-year windows reduced temporal mismatch but did not create stable cohorts: later windows contained fewer trials and greater leverage. Indication-specific prediction horizons are more defensible than assuming a cross-disease slope should strengthen automatically with follow-up.

Measurement error affected both axes. A published Cox HR summarizes observed follow-up and is not inherently a fixed-time effect, whereas curve-based approximations depend on image resolution, arm identification, numbers at risk, censoring, and proportional-hazards assumptions.^10^^,^^57^ Treating the early-endpoint estimate as error free can attenuate or distort the slope and understate uncertainty. Elevating the conditional errors-in-variables model to co-primary status directly addressed this concern. Its results remained imprecise across ρ assumptions, while some small-cohort profile bounds were not estimable; the data therefore do not identify a precise relationship under reasonable covariance choices.

The direct-HR-only analysis exposed a complementary problem. Removing reconstructed estimates improved source purity but reduced the estimable models to 3 or 4 trials, producing wide intervals and unstable prediction. Reconstruction therefore increases coverage at the cost of approximation, while restriction to directly reported estimates may introduce availability bias if mature or favorable results are more likely to be reported numerically. Agreement in the overall message across these analyses is reassuring only in a limited sense: neither data route supplied enough information to support surrogate validation.

The QoL findings require a benefit–risk rather than surrogacy interpretation. No strict-primary window reached 5 verified contrasts, so withholding regression avoided inferential weight on extreme 3-observation coefficients. The expanded verified-exact sensitivity remained imprecise. Global-health scores may have ceiling effects, while toxicity, recovery, recurrence prevention, and survivorship operate on different time scales. Heterogeneous windows, estimands, completion, and missing-data rules further weaken trial-level mapping between survival HRs and one QoL contrast.^7–9^ Lack of an estimable association is not evidence that QoL is unaffected or unimportant.

For clinical and trial-design use, these findings argue against replacing mature OS follow-up with a universal DFS-, RFS-, EFS-, or PFS-based prediction rule in adjuvant immunotherapy. Early endpoints may still be clinically meaningful direct outcomes, support accelerated decision making, and contribute to benefit–risk assessment when their definitions and effect sizes are compelling. However, their interpretation should remain indication specific, accompanied by continued OS follow-up and transparent reporting of subsequent therapies. The present analysis also should not be used to demand that every recurrence benefit already show an OS advantage; it addresses predictability across trials, not whether delaying recurrence benefits patients.

Future validation should use individual-patient data within disease-specific trial collections, harmonized definitions, prespecified horizons, and joint models incorporating treatment-effect covariance. Patient- and trial-level criteria, surrogate threshold effects, calibration, internal-external cross-validation, and external validation should be reported. Non-proportional hazards and competing risks require explicit handling. Trial reports should provide numerical HRs and CIs, covariance information or sufficient score statistics, numbers at risk, post-recurrence therapies, and arm-specific QoL completion. QoL protocols should prespecify domain, window, estimand, missing-data strategy, and the distinction between treatment burden and survivorship.

Strengths include restoration of the prespecified population, separation of DFS and RFS, a co-primary measurement-error model, explicit handling of PFS2, source-level HR labeling, exclusion of broad-taxonomy evidence, and a minimum-information rule for QoL. Limitations remain substantial: associations are ecological; later windows contain few trials; nested windows are dependent; reconstructed HRs are approximate; correlations were assumed; and disease, endpoint definition, OS maturity, and post-recurrence treatment remain heterogeneous. Reporting availability may also determine analyzable time points. The results are a boundary on current evidence and a guide for validation, not definitive proof for or against an endpoint within a specific cancer.

## Conclusion

Across the co-primary weighted and conditional errors-in-variables analyses, strict-primary DFS and RFS treatment effects did not show a consistent or precise aggregate relationship with OS. The principal measurement-error model did not resolve the uncertainty, and strict-primary QoL evidence was insufficient for regression. Eligibility-expanded, direct-HR-only, EFS, and PFS/PFS2 findings remain supplementary. These results do not validate DFS, RFS, EFS, or PFS as surrogates for OS.

## Supplementary Material

oyag342_Supplementary_Data

## Data Availability

All data analyzed in this study were derived from publicly available published studies and their associated supplementary materials. No new individual participant-level data were generated for this study. Additional data supporting the findings of this study, including the compiled study-level data used for the analyses, are available from the corresponding author upon reasonable request.
