# Correction: Linking early and late endpoints in adjuvant immunotherapy trials: an evaluation of multiple clinical outcomes

**DOI:** 10.1093/oncolo/oyag399

**Published:** 2026-10-14

This is a correction to: Sirui Tang, Huiran Zang, Linhan Mao, Xingyu Liu, Xinyu Li, Linking early and late endpoints in adjuvant immunotherapy trials: an evaluation of multiple clinical outcomes, *The Oncologist*, Volume 31, Issue 10, October 2026, oyag342, https://doi.org/10.1093/oncolo/oyag342

The affiliations have been corrected to be assigned and listed:

“Sirui Tang^1^, Huiran Zang^1^, Linhan Mao^2^, Xingyu Liu^3^, Xinyu Li^2^

^1^Department of Plastic Surgery, Peking University People’s Hospital, No. 11 Xizhimen South Street, Xicheng, Beijing, 100044, China

^2^Yunnan Normal University, Kunming, Yunnan, China

^3^University of Leeds, Leeds, United Kingdom”

Instead of:

“Sirui Tang^1^, Huiran Zang^1^, Linhan Mao^1^, Xingyu Liu^1^, Xinyu Li^1^

^1^Department of Plastic Surgery, Peking University People’s Hospital, No. 11 Xizhimen South Street, Xicheng, Beijing, 100044, China”.

