# Supplementary material for: Linking early and late endpoints in adjuvant immunotherapy trials: an evaluation of multiple clinical outcomes
Source: Oncologist. 2026 Aug 22;31(10):oyag342. doi: 10.1093/oncolo/oyag342 (PMC13584216; doi:10.1093/oncolo/oyag342)
Supplement: Supplementary file 1 [file oyag342_supplementary_data.zip › Figure S3.pdf]

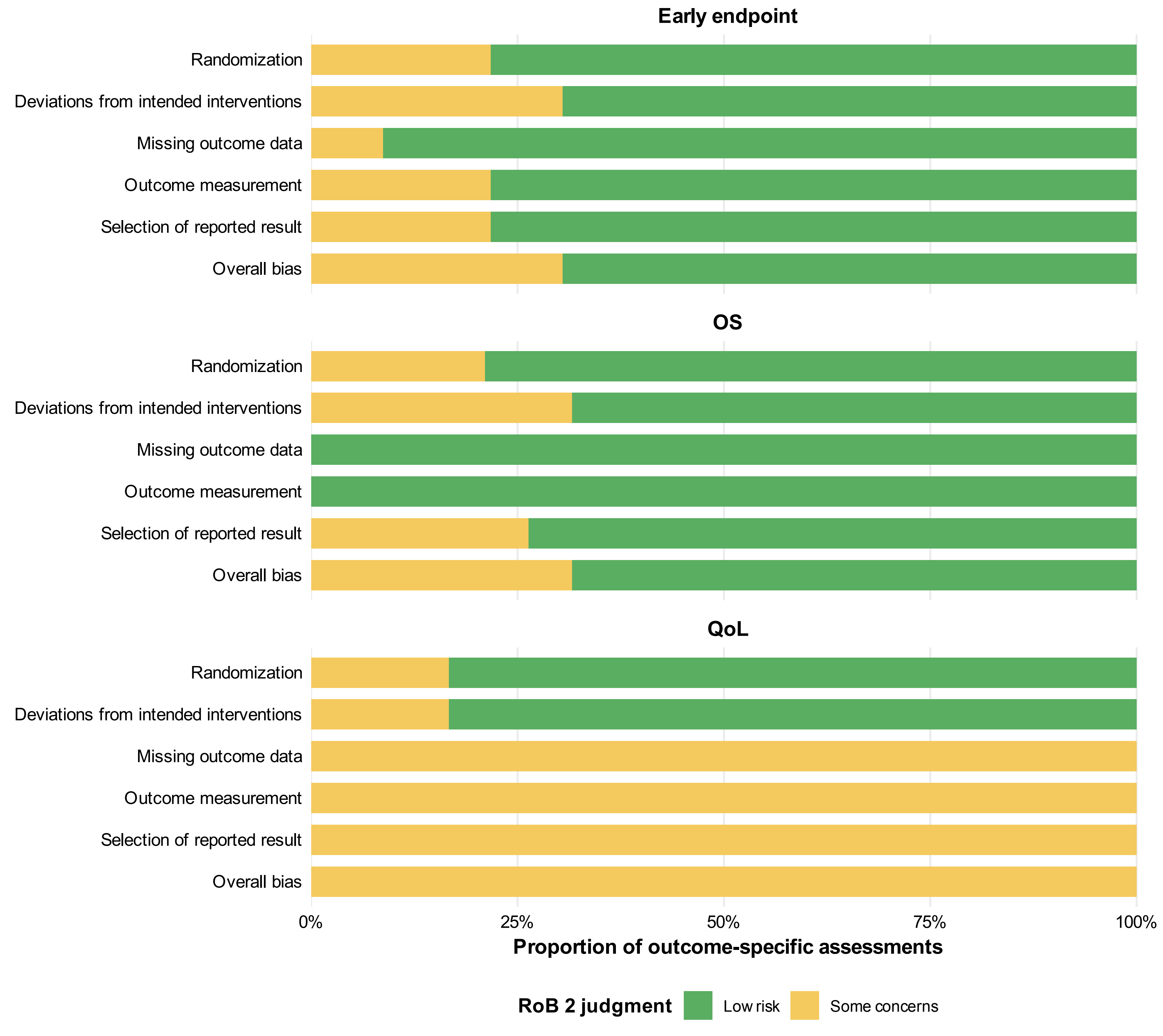

Early endpoint
Randomization
Deviations from intended interventions
Missing outcome data
Outcome measurement
Selection of reported result
Overall bias
OS
Randomization
Deviations from intended interventions
Missing outcome data
Outcome measurement
Selection of reported result
Overall bias
QoL
Randomization
Deviations from intended interventions
Missing outcome data
Outcome measurement
Selection of reported result
Overall bias
0%
25%
50%
75%
100%
Proportion of outcome-specific assessments
RoB 2 judgment
Low risk
Some concerns
