# Supplementary material for: Linking early and late endpoints in adjuvant immunotherapy trials: an evaluation of multiple clinical outcomes
Source: Oncologist. 2026 Aug 22;31(10):oyag342. doi: 10.1093/oncolo/oyag342 (PMC13584216; doi:10.1093/oncolo/oyag342)
Supplement: Supplementary file 1 [file oyag342_supplementary_data.zip › Figure S4.pdf]

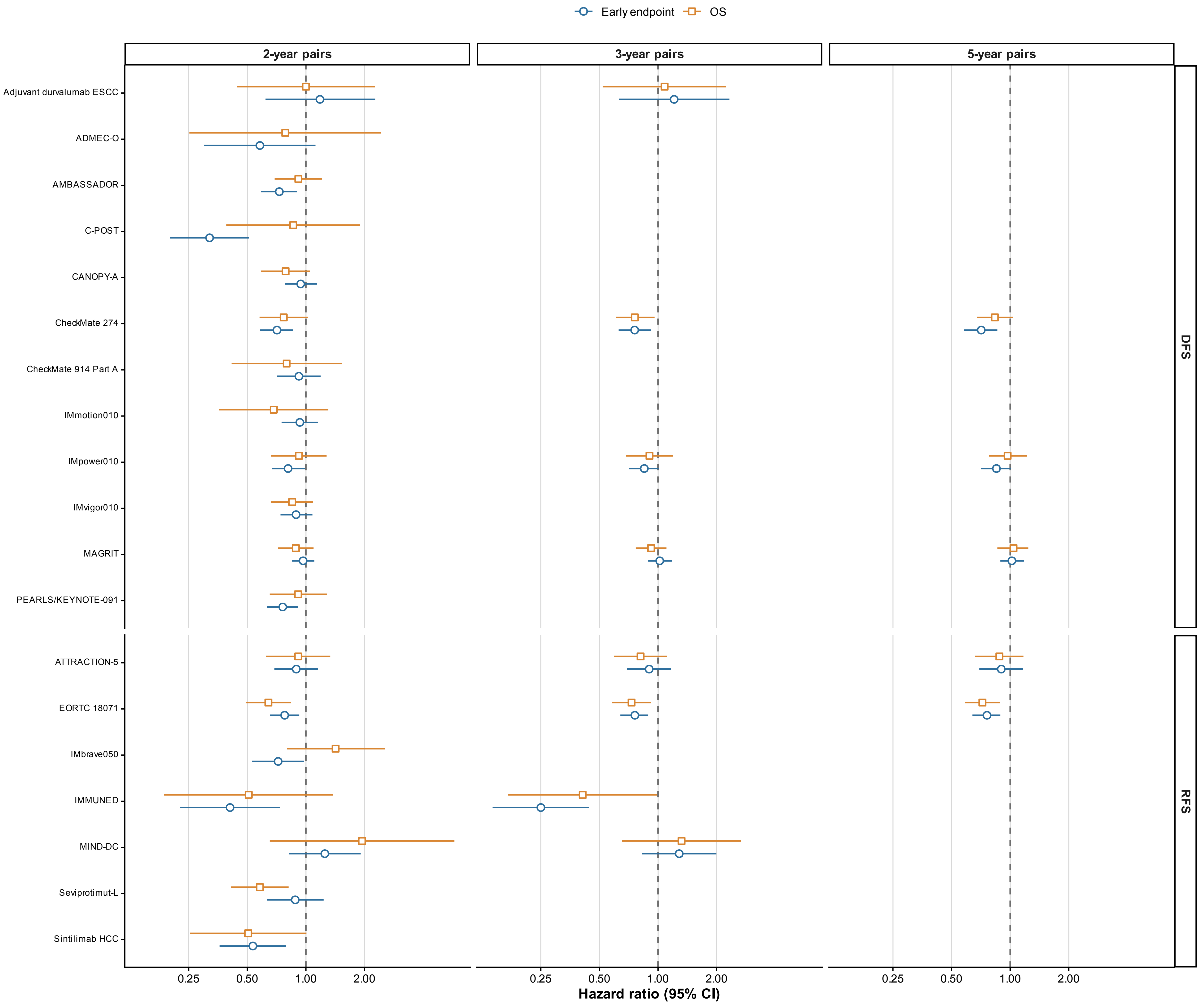

Early endpoint
OS
2-year pairs
3-year pairs
5-year pairs
Adjuvant durvalumab ESCC
ADMEC-O
AMBASSADOR
C-POST
CANOPY-A
CheckMate 274
CheckMate 914 Part A
IMmotion010
IMpower010
IMvigor010
MAGRIT
PEARLS/KEYNOTE-091
ATTRACTION-5
EORTC 18071
IMbrave050
IMMUNED
MIND-DC
Seviprotimut-L
Sintilimab HCC
DFS
RFS
0.25
0.50
1.00
2.00
Hazard ratio (95% CI)
