# Supplementary material for: Linking early and late endpoints in adjuvant immunotherapy trials: an evaluation of multiple clinical outcomes
Source: Oncologist. 2026 Aug 22;31(10):oyag342. doi: 10.1093/oncolo/oyag342 (PMC13584216; doi:10.1093/oncolo/oyag342)
Supplement: Supplementary file 1 [file oyag342_supplementary_data.zip › Figure S5.pdf]

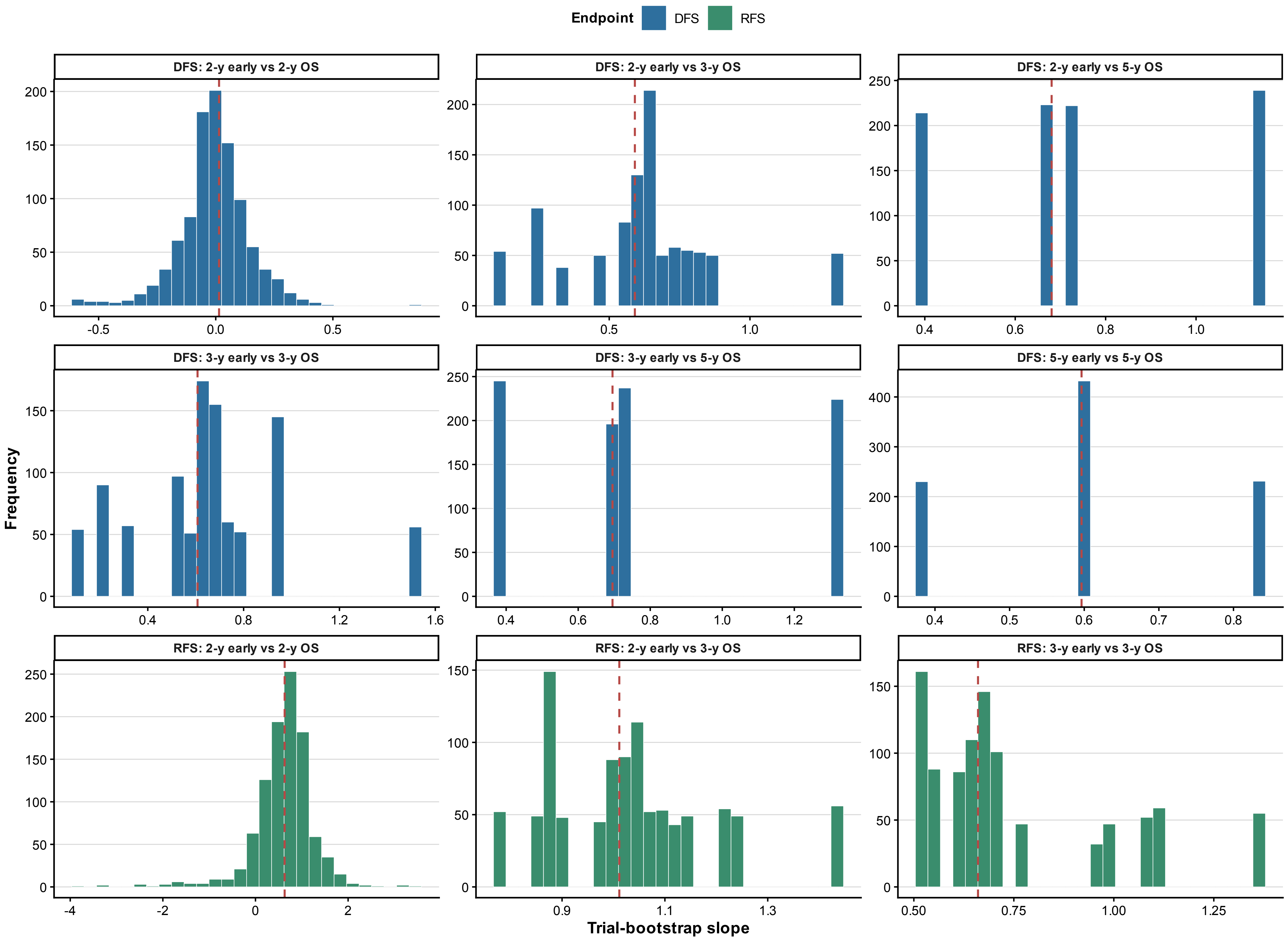

Endpoint
DFS
RFS
DFS: 2-y early vs 2-y OS
DFS: 2-y early vs 3-y OS
DFS: 2-y early vs 5-y OS
DFS: 3-y early vs 3-y OS
DFS: 3-y early vs 5-y OS
DFS: 5-y early vs 5-y OS
RFS: 2-y early vs 2-y OS
RFS: 2-y early vs 3-y OS
RFS: 3-y early vs 3-y OS
Frequency
Trial-bootstrap slope
