# Supplementary material for: Linking early and late endpoints in adjuvant immunotherapy trials: an evaluation of multiple clinical outcomes
Source: Oncologist. 2026 Aug 22;31(10):oyag342. doi: 10.1093/oncolo/oyag342 (PMC13584216; doi:10.1093/oncolo/oyag342)
Supplement: Supplementary file 1 [file oyag342_supplementary_data.zip › Figure S6.pdf]

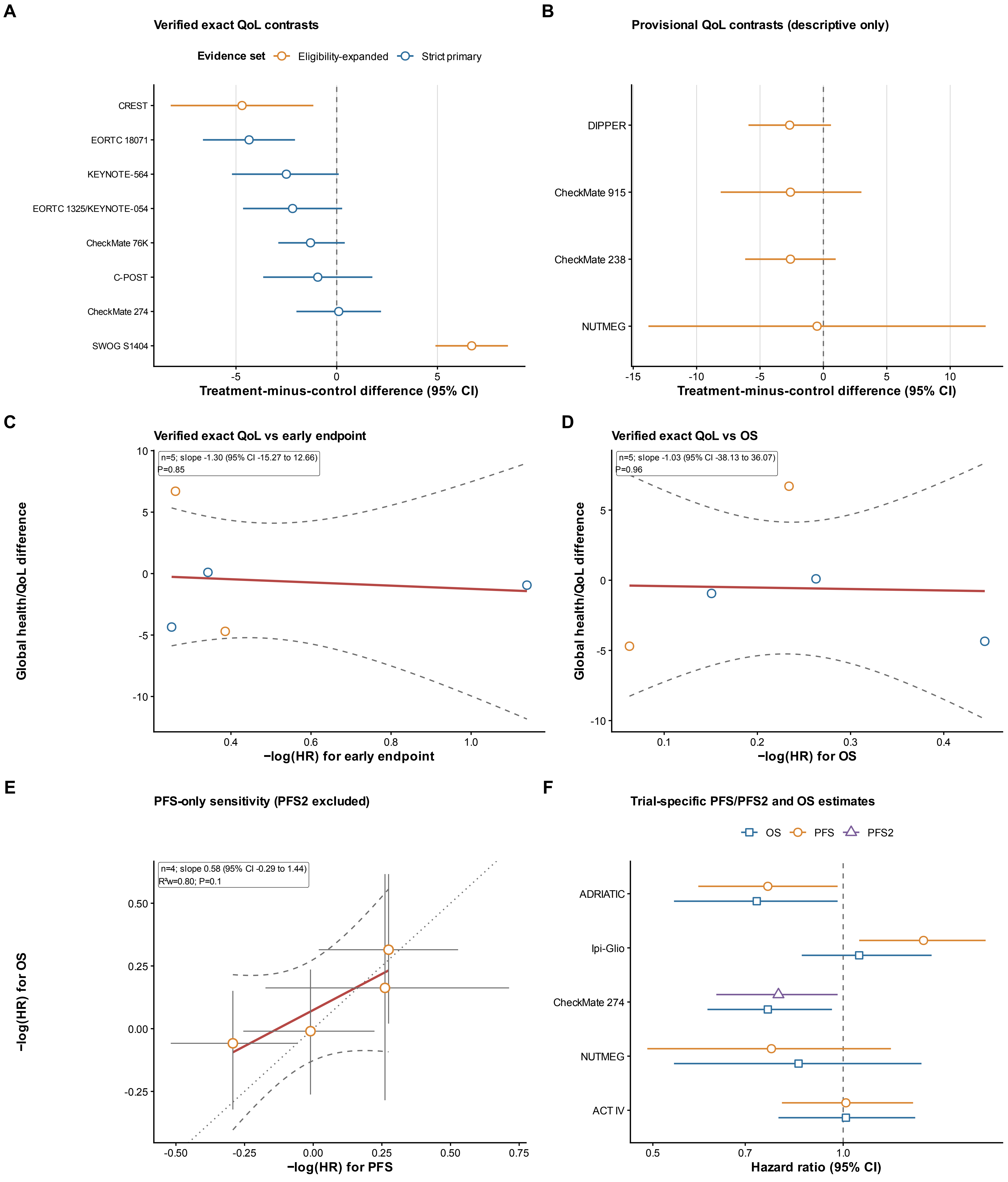

A
Verified exact QoL contrasts
Evidence set
Eligibility-expanded
Strict primary
CREST
EORTC 18071
KEYNOTE-564
EORTC 1325/KEYNOTE-054
CheckMate 76K
C-POST
CheckMate 274
SWOG S1404
Treatment-minus-control difference (95% CI)
B
Provisional QoL contrasts (descriptive only)
DIPPER
CheckMate 915
CheckMate 238
NUTMEG
Treatment-minus-control difference (95% CI)
C
Verified exact QoL vs early endpoint
n=5; slope -1.30 (95% CI -15.27 to 12.66)
P=0.85
Global health/QoL difference
−log(HR) for early endpoint
D
Verified exact QoL vs OS
n=5; slope -1.03 (95% CI -38.13 to 36.07)
P=0.96
Global health/QoL difference
−log(HR) for OS
E
PFS-only sensitivity (PFS2 excluded)
n=4; slope 0.58 (95% CI -0.29 to 1.44)
R²w=0.80; P=0.1
−log(HR) for OS
−log(HR) for PFS
F
Trial-specific PFS/PFS2 and OS estimates
OS
PFS
PFS2
ADRIATIC
Ipi-Glio
CheckMate 274
NUTMEG
ACT IV
Hazard ratio (95% CI)
