# Supplementary material for: Linking early and late endpoints in adjuvant immunotherapy trials: an evaluation of multiple clinical outcomes
Source: Oncologist. 2026 Aug 22;31(10):oyag342. doi: 10.1093/oncolo/oyag342 (PMC13584216; doi:10.1093/oncolo/oyag342)
Supplement: Supplementary file 1 [file oyag342_supplementary_data.zip › Figure S7.pdf]

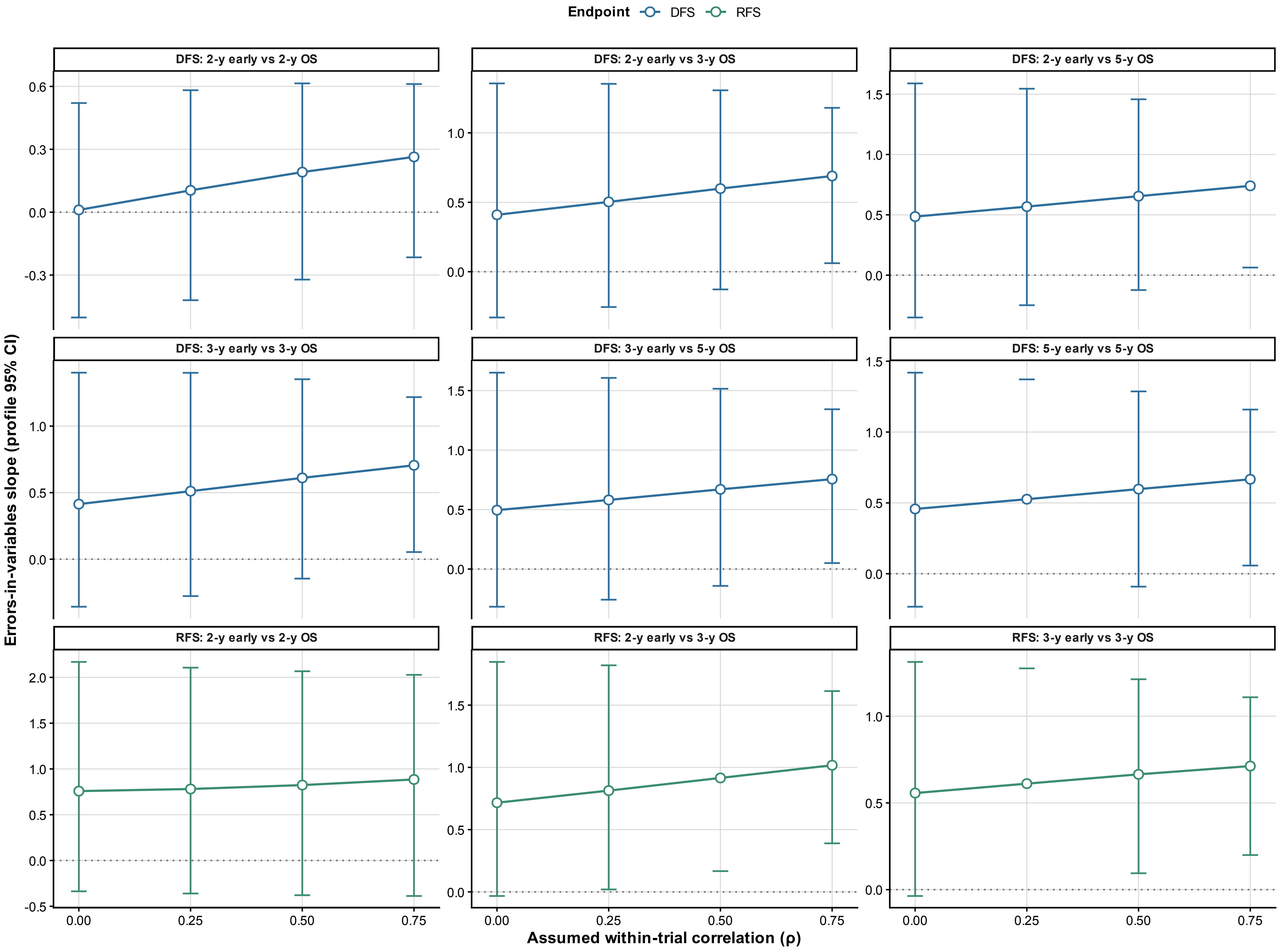

Endpoint DFS RFS
DFS: 2-y early vs 2-y OS
DFS: 2-y early vs 3-y OS
DFS: 2-y early vs 5-y OS
DFS: 3-y early vs 3-y OS
DFS: 3-y early vs 5-y OS
DFS: 5-y early vs 5-y OS
RFS: 2-y early vs 2-y OS
RFS: 2-y early vs 3-y OS
RFS: 3-y early vs 3-y OS
Errors-in-variables slope (profile 95% CI)
Assumed within-trial correlation (ρ)
0.00 0.25 0.50 0.75
