# Supplementary material for: Linking early and late endpoints in adjuvant immunotherapy trials: an evaluation of multiple clinical outcomes
Source: Oncologist. 2026 Aug 22;31(10):oyag342. doi: 10.1093/oncolo/oyag342 (PMC13584216; doi:10.1093/oncolo/oyag342)
Supplement: Supplementary file 1 [file oyag342_supplementary_data.zip › Figure S8.pdf]

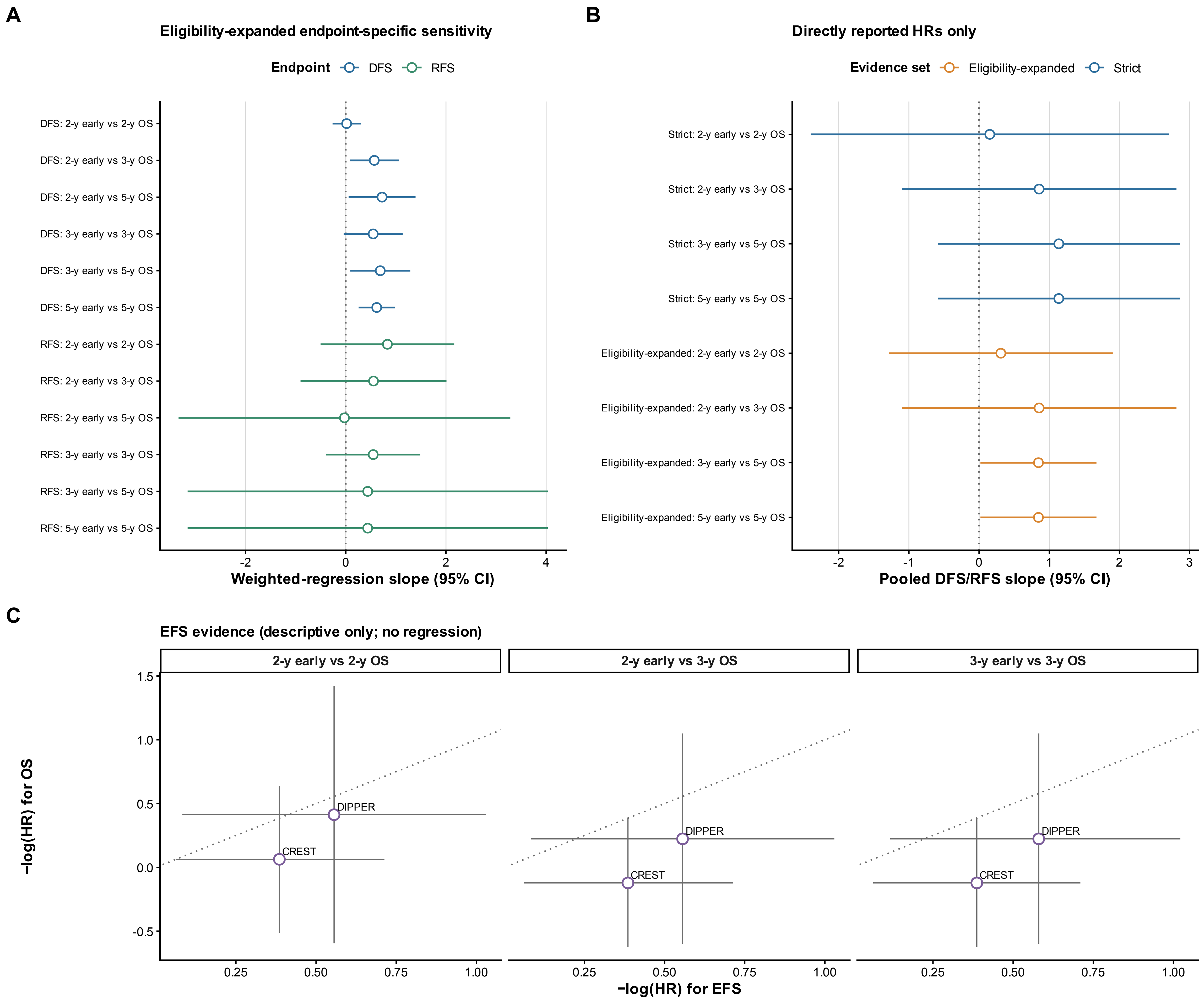
A
Eligibility-expanded endpoint-specific sensitivity
Endpoint DFS RFS
DFS: 2-y early vs 2-y OS
DFS: 2-y early vs 3-y OS
DFS: 2-y early vs 5-y OS
DFS: 3-y early vs 3-y OS
DFS: 3-y early vs 5-y OS
DFS: 5-y early vs 5-y OS
RFS: 2-y early vs 2-y OS
RFS: 2-y early vs 3-y OS
RFS: 2-y early vs 5-y OS
RFS: 3-y early vs 3-y OS
RFS: 3-y early vs 5-y OS
RFS: 5-y early vs 5-y OS
-2
0
2
4
Weighted-regression slope (95% CI)
B
Directly reported HRs only
Evidence set Eligibility-expanded Strict
Strict: 2-y early vs 2-y OS
Strict: 2-y early vs 3-y OS
Strict: 3-y early vs 5-y OS
Strict: 5-y early vs 5-y OS
Eligibility-expanded: 2-y early vs 2-y OS
Eligibility-expanded: 2-y early vs 3-y OS
Eligibility-expanded: 3-y early vs 5-y OS
Eligibility-expanded: 5-y early vs 5-y OS
-2
-1
0
1
2
3
Pooled DFS/RFS slope (95% CI)
C
EFS evidence (descriptive only; no regression)
2-y early vs 2-y OS
2-y early vs 3-y OS
3-y early vs 3-y OS
1.5
1.0
0.5
0.0
-0.5
−log(HR) for OS
DIPPER
CREST
0.25
0.50
0.75
1.00
−log(HR) for EFS
