# Supplementary material for: Linking early and late endpoints in adjuvant immunotherapy trials: an evaluation of multiple clinical outcomes
Source: Oncologist. 2026 Aug 22;31(10):oyag342. doi: 10.1093/oncolo/oyag342 (PMC13584216; doi:10.1093/oncolo/oyag342)
Supplement: Supplementary file 1 [file oyag342_supplementary_data.zip › Supplementary tables.docx]

**Protocol concordance statement**

The INPLASY record (INPLASY2025110071) was registered on 21 November 2025 and states that the review was completed but not published at registration; it is therefore described as retrospectively, not prospectively, registered. The protocol specified peer-reviewed English-language Phase II or III randomized trials published from 2016 onward in adults with resectable solid tumors receiving adjuvant or perioperative immunotherapy after curative-intent therapy, with a comparator that did not include immunotherapy. The strict primary set follows those core criteria. The 2015-only CIK HCC report, active-immunotherapy comparators, and nonoperative post-chemoradiotherapy settings are retained only as supplementary eligibility-sensitivity evidence. The broad-taxonomy study is descriptive only. EORTC 18071 qualified through its 2016 OS follow-up; its 2015 primary paper is retained as the original RFS source rather than treated as a separate pre-window trial. The originally proposed coauthor later requested withdrawal from the study. The first author subsequently changed institution and continued the research with the new research group. Accordingly, the registry retains the three protocol registrants and their registration-stage affiliation, whereas the final manuscript lists the five authors who contributed to the completed study and revision, all affiliated with Peking University People's Hospital. Both the registry and revised manuscript report no specific grant support.

**Table S1. Electronic database search strategies**

**PubMed Search Strategy**

((cancer[tiab] OR cancers[tiab] OR carcinoma*[tiab] OR tumor*[tiab] OR neoplasm*[tiab] OR malignan*[tiab])) AND ("immunotherapy"[tiab] OR "immune therapy"[tiab] OR "immune-based therap*"[tiab] OR "cancer vaccine*"[tiab] OR vaccin*[tiab] OR cytokine*[tiab] OR "interleukin-2"[tiab] OR IL-2[tiab] OR "interferon"[tiab] OR "T-cell therap*"[tiab] OR "cell-based immunotherapy"[tiab] OR "immune checkpoint inhibitor*"[tiab] OR ICI*[tiab] OR "PD-1"[tiab] OR "PD-L1"[tiab] OR "CTLA-4"[tiab] OR pembrolizumab OR nivolumab OR atezolizumab OR durvalumab OR avelumab OR ipilimumab OR cemiplimab OR toripalimab OR camrelizumab OR sintilimab OR tislelizumab) AND (adjuvant[tiab] OR postoperative[tiab] OR perioperative[tiab] OR resection*[tiab]) AND (randomized controlled trial[pt] OR randomized[tiab] OR randomised[tiab] OR RCT[tiab]) AND ("recurrence-free survival"[tiab] OR RFS[tiab] OR "disease-free survival"[tiab] OR DFS[tiab] OR "event-free survival"[tiab] OR EFS[tiab] OR "progression-free survival"[tiab] OR PFS[tiab] OR "overall survival"[tiab] OR OS[tiab] OR "quality of life"[tiab] OR QOL[tiab] OR "EORTC QLQ-C30"[tiab])

**Embase Search Strategy**

('cancer'/exp OR cancer*:ti,ab OR tumor*:ti,ab OR neoplasm*:ti,ab) AND('immunotherapy'/exp OR immunotherapy:ti,ab OR "immune-based therap*":ti,ab OR "immune therap*":ti,ab OR "cancer vaccine*":ti,ab OR vaccin*:ti,ab OR cytokine*:ti,ab OR "interleukin-2":ti,ab OR IL-2:ti,ab OR interferon*:ti,ab OR "cell-based immunotherapy":ti,ab OR "T-cell therap*":ti,ab OR "immune checkpoint inhibitor"/exp OR "immune checkpoint inhibitor*":ti,ab OR ICI*:ti,ab OR PD-1:ti,ab OR PD-L1:ti,ab OR CTLA-4:ti,ab OR pembrolizumab:ti,ab OR nivolumab:ti,ab OR atezolizumab:ti,ab OR durvalumab:ti,ab OR avelumab:ti,ab OR ipilimumab:ti,ab OR camrelizumab:ti,ab OR toripalimab:ti,ab OR sintilimab:ti,ab OR tislelizumab:ti,ab) AND (adjuvant:ti,ab OR postoperative:ti,ab OR perioperative:ti,ab OR resection*:ti,ab) AND (randomized controlled trial/exp OR randomized:ti,ab OR randomised:ti,ab OR RCT:ti,ab) AND (

"recurrence-free survival":ti,ab OR RFS:ti,ab OR DFS:ti,ab OR EFS:ti,ab OR PFS:ti,ab OR "overall survival":ti,ab OR OS:ti,ab OR "quality of life":ti,ab OR QOL:ti,ab OR "QLQ-C30":ti,ab)

**Cochrane Library Search Strategy**

(cancer* OR tumor* OR tumour* OR neoplasm* OR malignan*) AND (immunotherapy OR "immune therapy" OR "immune-based therap*" OR "cancer vaccine*" OR vaccin* OR cytokine* OR "interleukin-2" OR IL-2 OR interferon* OR "cell-based immunotherapy" OR "T-cell therap*"

OR "immune checkpoint inhibitor*" OR ICI* OR PD-1 OR PD-L1 OR CTLA-4 OR pembrolizumab OR nivolumab OR ipilimumab OR atezolizumab OR durvalumab OR avelumab OR camrelizumab OR toripalimab OR sintilimab OR tislelizumab) AND (adjuvant OR postoperative OR perioperative OR resection*) AND("recurrence-free survival" OR RFS OR DFS OR EFS OR PFS OR "overall survival" OR OS OR "quality of life" OR QOL OR "QLQ-C30")

**Web of Science Search Strategy**

TS=((cancer* OR tumor* OR tumour* OR neoplasm* OR malignan*) AND (immunotherapy OR "immune therapy" OR "immune-based therap*" OR "cancer vaccine*" OR vaccin* OR cytokine* OR "interleukin-2" OR IL-2 OR interferon* OR "cell-based immunotherapy" OR "T-cell therap*"

OR "immune checkpoint inhibitor*" OR ICI* OR PD-1 OR PD-L1 OR CTLA-4 OR pembrolizumab OR nivolumab OR ipilimumab OR atezolizumab OR durvalumab OR avelumab OR camrelizumab OR sintilimab OR tislelizumab OR toripalimab) AND (adjuvant OR postoperative OR perioperative OR resection*) AND (randomized OR randomised OR "randomized controlled trial" OR RCT) AND("recurrence-free survival" OR RFS OR DFS OR EFS OR PFS OR "overall survival" OR OS OR "quality of life" OR QOL OR "QLQ-C30"))

**Table S2. Complete audit trail, characteristics, endpoint definitions, and extracted estimates**

**Part A. Disposition of all 44 submitted source records**

| **Raw row** | **Submitted registration** | **Submitted first author/year** | **Submitted record** | **Final disposition** | **Canonical trial/registration** | **Audit rationale** |
| --- | --- | --- | --- | --- | --- | --- |
| 1 | NCT02628067 | Watowich M, 2024 | Phase II Trial Evaluating the Association of Peripheral Blood Immunologic Response to Therapeutic Response to Adjuvant Treatment With Immune C… | Excluded | — | nonrandomized advanced KEYNOTE-158/mismatched intervention |
| 2 | NCT02486718 | E. Felip, 2023/2024 | Overall survival with adjuvant atezolizumab after chemotherapy in resected stage II-IIIA non-small-cell lung cancer (IMpower010): a randomised… | Strict primary | IMpower010 (NCT02486718) | Correct full randomized trial; retains stage II-IIIA efficacy population used in the source paper. |
| 3 | NCT03703297 | Ying Cheng, 2024 | Durvalumab after Chemoradiotherapy in Limited-Stage Small-Cell Lung Cancer | Expanded sensitivity | ADRIATIC (NCT03703297) | Nonoperative post-chemoradiotherapy consolidation setting. |
| 4 | ISRCTN84434175 | Nicholas F Brown, 2025 | Ipilimumab with temozolomide vs. temozolomide alone after surgery and chemoradiotherapy in recently diagnosed glioblastoma: a randomized phase… | Expanded sensitivity | Ipi-Glio (ISRCTN84434175) | Glioblastoma treatment begins after surgery and chemoradiotherapy; not a conventional adjuvant-only setting. |
| 5 | NCT04165317 | Not recorded, 2025 | Sasanlimab plus BCG in BCG-naive, high-risk non-muscle invasive bladder cancer: the randomized phase 3 CREST trial | Expanded sensitivity | CREST (NCT04165317) | BCG is an active immunotherapy comparator in both randomized groups. |
| 6 | NCT02388906 | Paolo A Ascierto, 2025 | Nivolumab for Resected Stage III or IV Melanoma at 9 Years | Expanded sensitivity | CheckMate 238 (NCT02388906) | Ipilimumab is an active immunotherapy comparator. |
| 7 | NCT03095040 | Xinan Sheng, 2022 | The Efficacy and Safety of Vorolanib Monotherapy or in Combination With Toripalimab for Postoperative Adjuvant Treatment of Renal Cell Carcino… | Excluded | — | metastatic second-line RCC, not adjuvant |
| 8 | NCT02196961 | J.C. Becker, 2022 | Adjuvant immunotherapy with nivolumab (NIVO) versus observation in completely resected Merkel cell carcinoma (MCC): Disease-free survival (DFS… | Strict primary | ADMEC-O (NCT02196961) | Peer-reviewed full paper replaces the conference-abstract citation in the old dataset. |
| 9 | NCT00636168 | Alexander M M Eggermont, 2016 | Prolonged Survival in Stage III Melanoma with Ipilimumab Adjuvant Therapy | Strict primary | EORTC 18071 (NCT00636168) | Rows 9 and 18 are one trial. Row 18 carried an incorrect registry identifier and is merged here. |
| 10 | NCT02632409 | Matthew D Galsky, 2025 | Adjuvant Nivolumab in High-Risk Muscle-Invasive Urothelial Carcinoma: Expanded Efficacy From CheckMate 274 | Strict primary | CheckMate 274 (NCT02632409) | Old QoL confidence interval is internally impossible and is corrected from the primary QoL paper. |
| 11 | NCT02506153 | Kenneth F Grossmann, 2022 | Adjuvant Pembrolizumab versus IFNα2b or Ipilimumab in Resected High-Risk Melanoma | Expanded sensitivity | SWOG S1404 (NCT02506153) | Ipilimumab/high-dose interferon is an active control; comparator differs from placebo/observation. |
| 12 | NCT01176474 | Nikhil I Khushalani, 2022 | Phase II clinical and immune correlate study of adjuvant nivolumab plus ipilimumab for high-risk resected melanoma | Excluded | — | phase I, nonrandomized, no eligible comparator |
| 13 | NCT04781413 | Yu Mei, 2021 | Addition of sintilimab to nanoparticle albumin-bound paclitaxel and S-1 as adjuvant therapy in stage IIIC gastric cancer | Excluded | — | single-arm phase I/II protocol and unsupported N/comparator |

**Table S2. Part A (continued)**

| **Raw row** | **Submitted registration** | **Submitted first author/year** | **Submitted record** | **Final disposition** | **Canonical trial/registration** | **Audit rationale** |
| --- | --- | --- | --- | --- | --- | --- |
| 14 | NCT03142334 | Se Hoon Park, 2025 | Adjuvant Pembrolizumab Versus Placebo for Renal Cell Carcinoma in the East Asian Subgroup of the Phase 3 KEYNOTE-564 Study | Strict primary after source replacement | KEYNOTE-564 (NCT03142334) | Replace subgroup publication by full KEYNOTE-564 trial |
| 15 | NCT02450331 | Joaquim Bellmunt, 2024 | Adjuvant atezolizumab versus observation in muscle-invasive urothelial carcinoma (IMvigor010): a multicentre, open-label, randomised, phase 3… | Strict primary | IMvigor010 (NCT02450331) | Corrected analysis population; old workbook used a smaller unsupported N. |
| 16 | NCT01274338 | Ahmad A Tarhini, 2020 | Phase III Study of Adjuvant Ipilimumab (3 or 10 mg/kg) Versus High-Dose Interferon Alfa-2b for Resected High-Risk Melanoma: North American Int… | Expanded sensitivity | E1609 (NCT01274338) | High-dose interferon is an active immunotherapy comparator. |
| 17 | NCT04195139 | Hao-Wen Sim, 2023 | NUTMEG: A randomized phase II study of nivolumab and temozolomide versus temozolomide alone in newly diagnosed older patients with glioblastoma | Expanded sensitivity | NUTMEG (NCT04195139) | Glioblastoma regimen after chemoradiotherapy; not a conventional adjuvant-only setting. |
| 18 | NCT02970981 | Alexander M M Eggermont, 2019 | Adjuvant ipilimumab versus placebo after complete resection of stage III melanoma: long-term follow-up results of the European Organisation fo… | Duplicate/merged | EORTC 18071 (NCT00636168) | Merge duplicate into EORTC 18071 and correct registry ID |
| 19 | NCT03178123 | B Lian, 2022 | Toripalimab (anti-PD-1) versus high-dose interferon-α2b as adjuvant therapy in resected mucosal melanoma: a phase II randomized trial | Expanded sensitivity | Toripalimab vs HDI (NCT03178123) | High-dose interferon is an active immunotherapy comparator. |
| 20 | NCT03528694 | Prof Maria De Santis, MD, 2025 | Durvalumab in combination with BCG for BCG-naive, high-risk, non-muscle-invasive bladder cancer (POTOMAC): final analysis of a randomised, ope… | Expanded sensitivity | POTOMAC (NCT03528694) | BCG is an active immunotherapy comparator in both randomized groups. |
| 21 | NCT02504372 | Mary O'Brien, 2022 | Pembrolizumab versus placebo as adjuvant therapy for completely resected stage IB-IIIA non-small-cell lung cancer (PEARLS/KEYNOTE-091): an int… | Strict primary | PEARLS/KEYNOTE-091 (NCT02504372) | Eligible after registry and publication concordance audit. |
| 22 | NCT03024996 | Sumanta Kumar Pal, 2022 | Adjuvant atezolizumab versus placebo for patients with renal cell carcinoma at increased risk of recurrence following resection (IMmotion010):… | Strict primary | IMmotion010 (NCT03024996) | Eligible after registry and publication concordance audit. |
| 23 | NCT04102098 | Shukui Qin, 2023 | Atezolizumab plus bevacizumab versus active surveillance in patients with resected or ablated high-risk hepatocellular carcinoma (IMbrave050):… | Strict primary | IMbrave050 (NCT04102098) | Uses the publication available in the submitted dataset; post-submission updates are not introduced here. |
| 24 | NCT03969004 | Danny Rischin, 2025 | Adjuvant Cemiplimab or Placebo in High-Risk Cutaneous Squamous-Cell Carcinoma | Strict primary | C-POST (NCT03969004) | QoL report is conference-only and is excluded from the peer-reviewed QoL regression. |
| 25 | NCT03244384 | Andrea B Apolo, 2025 | Adjuvant Pembrolizumab versus Observation in Muscle-Invasive Urothelial Carcinoma | Strict primary | AMBASSADOR (NCT03244384) | Eligible after registry and publication concordance audit. |

**Table S2. Part A (continued)**

| **Raw row** | **Submitted registration** | **Submitted first author/year** | **Submitted record** | **Final disposition** | **Canonical trial/registration** | **Audit rationale** |
| --- | --- | --- | --- | --- | --- | --- |
| 26 | ChiCTR2000037655 | Kang Wang, 2024 | Adjuvant sintilimab in resected high-risk hepatocellular carcinoma: a randomized, controlled, phase 2 trial | Strict primary | Sintilimab HCC (ChiCTR2000037655) | Chinese registry record; endpoint wording is retained from the primary paper until a machine-readable registry export is archived. |
| 27 | NCT02317471 | Kecheng Zhang, 2017 | Phase II Trial of Adjuvant Immunotherapy with Autologous Tumor-derived Gp96 Vaccination in Patients with Gastric Cancer | Excluded | — | explicitly nonrandomized gp96 study |
| 28 | NCT02125461 | Jarushka Naidoo, MbChB., MHS, 2023 | Brief Report: Durvalumab After Chemoradiotherapy in Unresectable Stage III EGFR-Mutant NSCLC: A Post Hoc Subgroup Analysis From PACIFIC | Excluded | — | PACIFIC unresectable EGFR post hoc subgroup |
| 29 | NCT03068455 | Jeffrey S Weber, 2023 | Adjuvant Therapy of Nivolumab Combined With Ipilimumab Versus Nivolumab Alone in Patients With Resected Stage IIIB-D or Stage IV Melanoma (Che… | Expanded sensitivity | CheckMate 915 (NCT03068455) | Nivolumab is an active immunotherapy comparator. |
| 30 | NCT01480479 | Michael Weller, 2017 | Rindopepimut with temozolomide for patients with newly diagnosed, EGFRvIII-expressing glioblastoma (ACT IV): a randomised, double-blind, inter… | Strict primary | ACT IV (NCT01480479) | Eligible after registry and publication concordance audit. |
| 31 | NCT00480025 | Prof Dr Johan F Vansteenkiste, 2016 | xfEfficacy of the MAGE-A3 cancer immunotherapeutic as adjuvant therapy in patients with resected MAGE-A3-positive non-small-cell lung cancer (… | Strict primary | MAGRIT (NCT00480025) | Corrects the old workbook N, which did not represent the randomized efficacy population. |
| 32 | NCT01546571 | Craig L Slingluff, 2021 | Multicenter, double-blind, placebo-controlled trial of seviprotimut-L polyvalent melanoma vaccine in patients with post-resection melanoma at… | Strict primary | Seviprotimut-L (NCT01546571) | Eligible after registry and publication concordance audit. |
| 33 | NCT00699816 | Joon Hyeok Lee, 2016 | Adjuvant immunotherapy with autologous cytokine-induced killer cells for hepatocellular carcinoma | Expanded sensitivity | CIK HCC (NCT00699816) | Primary paper was published in 2015, before the protocol publication window. |
| 34 | NCT02520453 | S Park, 2022 | Adjuvant durvalumab for esophageal squamous cell carcinoma after neoadjuvant chemoradiotherapy: a placebo-controlled, randomized, double-blind… | Strict primary | Adjuvant durvalumab ESCC (NCT02520453) | Eligible after registry and publication concordance audit. |
| 35 | NCT02523313 | Elisabeth Livingstone, 2022 | Adjuvant nivolumab plus ipilimumab or nivolumab alone versus placebo in patients with resected stage IV melanoma with no evidence of disease (… | Strict primary | IMMUNED (NCT02523313) | Eligible after registry and publication concordance audit. |
| 36 | NCT00819650 | Jun Li, 2020 | Adjuvant ¹³¹I-metuximab for hepatocellular carcinoma after liver resection: a randomised, controlled, multicentre, open-label, phase 2 trial | Broad taxonomy sensitivity | 131I-metuximab HCC (NCT00819650) | Radioimmunotherapy/targeted-radioisotope mechanism is outside the core immunotherapy taxonomy. |
| 37 | NCT03006705 | Yoon-Koo Kang, 2024 | Adjuvant nivolumab plus chemotherapy versus placebo plus chemotherapy for stage III gastric or gastro-oesophageal junction cancer after gastre… | Strict primary | ATTRACTION-5 (NCT03006705) | Eligible after registry and publication concordance audit. |

**Table S2. Part A (continued)**

| **Raw row** | **Submitted registration** | **Submitted first author/year** | **Submitted record** | **Final disposition** | **Canonical trial/registration** | **Audit rationale** |
| --- | --- | --- | --- | --- | --- | --- |
| 38 | NCT03138512 | Robert J Motzer, 2023 | Adjuvant nivolumab plus ipilimumab versus placebo for localised renal cell carcinoma after nephrectomy (CheckMate 914): results from a double-… | Strict primary | CheckMate 914 Part A (NCT03138512) | Part A is retained as the comparison represented in the submitted dataset. |
| 39 | NCT02993315 | Kalijn F Bol, 2024 | Adjuvant dendritic cell therapy in stage IIIB/C melanoma: the MIND-DC randomized phase III trial | Strict primary | MIND-DC (NCT02993315) | Eligible after registry and publication concordance audit. |
| 40 | NCT03427827 | Ye-Lin Liang, 2025 | Adjuvant PD-1 Blockade With Camrelizumab for Nasopharyngeal Carcinoma | Expanded sensitivity | DIPPER (NCT03427827) | Post-chemoradiotherapy nasopharyngeal carcinoma setting rather than surgery-based adjuvant therapy. |
| 41 | NCT03447769 | Edward B. Garon, 2023 | Canakinumab as Adjuvant Therapy in Patients With Completely Resected Non–Small-Cell Lung Cancer: Results From the CANOPY-A Double-Blind, Rando… | Strict primary | CANOPY-A (NCT03447769) | Eligible after registry and publication concordance audit. |
| 42 | NCT00324805 | Heather A Wakelee, 2017 | Adjuvant chemotherapy with or without bevacizumab in patients with resected non-small-cell lung cancer (E1505): an open-label, multicentre, ra… | Excluded | — | bevacizumab targeted therapy, outside protocol taxonomy |
| 43 | NCT02362594 | Oliver John Kennedy, 2024 | Prognostic and predictive value of non-steroidal anti-inflammatory drugs in the EORTC 1325/KEYNOTE-054 phase III trial of pembrolizumab versus… | Strict primary after source replacement | EORTC 1325/KEYNOTE-054 (NCT02362594) | Replace NSAID secondary analysis by primary KEYNOTE-054 sources |
| 44 | NCT04099251 | John M Kirkwood, 2024/2025 | Adjuvant nivolumab in resected stage IIB/C melanoma: primary results from the randomized, phase 3 CheckMate 76K trial | Strict primary | CheckMate 76K (NCT04099251) | Eligible after registry and publication concordance audit. |

The 44 entries are source records, not 44 independent eligible trials. After correction of duplicate, subgroup, and mismatched records, the strict primary set contains 23 unique trials; 12 additional trials are retained as eligibility-sensitivity evidence, one as broad immunotherapy-taxonomy evidence, and seven records are excluded. The 23-trial strict set defines the main quantitative analysis. Eligibility-sensitivity studies are used only in supplementary sensitivity analyses, and the broad-taxonomy trial is retained only in the descriptive audit ledger.

**Part B1. Characteristics and publications for the 23 strict-set trials**

| **Trial** | **Registration** | **Disease/stage** | **Phase/randomized N** | **Intervention** | **Comparator** | **Primary/OS publications** | **QoL publication(s)** |
| --- | --- | --- | --- | --- | --- | --- | --- |
| IMpower010 | NCT02486718 | non-small-cell lung cancer; stage II-IIIA | Phase III; N=1005 | atezolizumab | BSC after adjuvant platinum based chemotherapy | Primary efficacy: 34555333 (2021); OS follow-up: 37467930 (2023); Long-term efficacy: 40446184 (2025) | None identified in the strict peer-reviewed dataset. |
| ADMEC-O | NCT02196961 | Merkel cell carcinoma; stage I/II vs. III/IV | Phase II; N=179 | nivolumab | observation | Primary efficacy: 37451295 (2023) | None identified in the strict peer-reviewed dataset. |
| EORTC 18071 | NCT00636168 | melanoma; stage Ⅲ | Phase III; N=951 | ipilimumab | placebo | Primary efficacy: 25840693 (2015); OS follow-up: 27717298 (2016) | QoL: 28162999 (2017) |
| CheckMate 274 | NCT02632409 | Muscle-Invasive Urothelial Carcinoma | Phase III; N=709 | nivolumab | placebo | Primary efficacy: 34077643 (2021); Expanded efficacy: 39393026 (2025) | QoL: 35288066 (2022) |
| KEYNOTE-564 | NCT03142334 | Renal Cell Carcinoma | Phase III; N=994 | pembrolizumab | placebo | Primary efficacy: 34407342 (2021); OS follow-up: 38631003 (2024) | QoL: 37589219 (2024) |
| IMvigor010 | NCT02450331 | Muscle-invasive Urothelial Carcinoma | Phase III; N=809 | atezolizumab | observation | Primary efficacy: 33721560 (2021); OS follow-up: 37500339 (2024) | None identified in the strict peer-reviewed dataset. |
| PEARLS/KEYNOTE-091 | NCT02504372 | non-small-cell lung cancer; stage 1b\2\3a | Phase III; N=1177 | pembrolizumab | placebo | Primary efficacy: 36108662 (2022) | None identified in the strict peer-reviewed dataset. |
| IMmotion010 | NCT03024996 | renal cell carcinoma | Phase III; N=778 | atezolizumab | placebo | Primary efficacy: 36099926 (2022) | None identified in the strict peer-reviewed dataset. |
| IMbrave050 | NCT04102098 | hepatocellular carcinoma | Phase III; N=668 | atezolizumab plus bevacizumab | active surveillance | Primary efficacy: 37871608 (2023) | None identified in the strict peer-reviewed dataset. |
| C-POST | NCT03969004 | Cutaneous Squamous-Cell Carcinoma | Phase III; N=415 | cemiplimab | placebo | Primary efficacy: 40454639 (2025) | None identified in the strict peer-reviewed dataset. |
| AMBASSADOR | NCT03244384 | Muscle-Invasive Urothelial Carcinoma; stage 3 | Phase III; N=702 | pembrolizumab | observation | Primary efficacy: 39282902 (2025) | None identified in the strict peer-reviewed dataset. |
| Sintilimab HCC | ChiCTR2000037655 | hepatocellular carcinoma | Phase II; N=198 | adjuvant sintilimab | active surveillance | Primary efficacy: 38242982 (2024) | None identified in the strict peer-reviewed dataset. |
| ACT IV | NCT01480479 | glioblastoma | Phase III; N=745 | rindopepimut/GM-CSF plus temozolomide | KLH control plus temozolomide | Primary efficacy: 28844499 (2017) | None identified in the strict peer-reviewed dataset. |
| MAGRIT | NCT00480025 | NSCLC | Phase III; N=2272 | MAGE-A3 immunotherapeutic | placebo | Primary efficacy: 27132212 (2016) | None identified in the strict peer-reviewed dataset. |
| Seviprotimut-L | NCT01546571 | melanoma; stage IIB-III | Phase III; N=347 | seviprotimut-L | placebo | Primary efficacy: 34599031 (2021) | None identified in the strict peer-reviewed dataset. |
| Adjuvant durvalumab ESCC | NCT02520453 | esophageal squamous cell carcinoma | Phase II; N=86 | durvalumab | placebo | Primary efficacy: 35158205 (2022) | None identified in the strict peer-reviewed dataset. |
| IMMUNED | NCT02523313 | melanoma; stage 4 | Phase II; N=167 | nivolumab plus ipilimumab/nivolumab | placebo | Primary efficacy: 32416781 (2020); Final efficacy: 36099927 (2022) | None identified in the strict peer-reviewed dataset. |
| ATTRACTION-5 | NCT03006705 | gastric or gastro-oesophageal junction cancer; stage 3 | Phase III; N=755 | nivolumab plus chemotherapy | placebo plus chemotherapy | Primary efficacy: 38906161 (2024) | None identified in the strict peer-reviewed dataset. |
| CheckMate 914 Part A | NCT03138512 | renal cell carcinoma | Phase III; N=816 | nivolumab plus ipilimumab | placebo | Primary efficacy: 36774933 (2023) | None identified in the strict peer-reviewed dataset. |
| MIND-DC | NCT02993315 | melanoma; stage ⅢB-C | Phase III; N=148 | autologous natural dendritic-cell vaccination | placebo | Primary efficacy: 38395969 (2024) | None identified in the strict peer-reviewed dataset. |
| CANOPY-A | NCT03447769 | Non–Small-Cell Lung Cancer; stage II-IIIA or IIIB | Phase III; N=1382 | canakinumab | placebo | Primary efficacy: 37788412 (2024) | None identified in the strict peer-reviewed dataset. |
| EORTC 1325/KEYNOTE-054 | NCT02362594 | melanoma; stage Ⅲ | Phase III; N=1019 | pembrolizumab | placebo | Primary efficacy: 29658430 (2018); Five-year efficacy: 38319852 (2022) | QoL: 33857414 (2021); Long-term QoL: 39146951 (2024) |
| CheckMate 76K | NCT04099251 | melanoma; stage IIB/C | Phase III; N=790 | nivolumab | placebo | Primary efficacy: 37845511 (2023) | QoL: 40139004 (2025) |

Publication identifiers refer to the exact primary, updated efficacy/OS, and QoL reports used for source verification. Full titles, journals, dates, DOIs, and source URLs are retained in Canonical trial data.xlsx.

**Part B2. Characteristics of 12 eligibility-sensitivity and one broad-taxonomy trial**

| **Trial** | **Evidence set** | **Registration** | **Disease/stage** | **Phase/randomized N** | **Intervention** | **Comparator** | **Primary/OS publications** | **Reason outside strict quantitative set** |
| --- | --- | --- | --- | --- | --- | --- | --- | --- |
| ADRIATIC | Expanded sensitivity | NCT03703297 | small-cell lung cancer; stage I or II vs. III | Phase III; N=730 | durvalumab/durvalumab-tremelimumab | placebo | Primary efficacy: 39268857 (2024) | Nonoperative post-chemoradiotherapy consolidation setting. |
| Ipi-Glio | Expanded sensitivity | ISRCTN84434175 | Glioblastoma | Phase II; N=129 | ipilimumab + temozolomide | temozolomide | Primary efficacy: 40800123 (2025) | Glioblastoma treatment begins after surgery and chemoradiotherapy; not a conventional adjuvant-only setting. |
| CREST | Expanded sensitivity | NCT04165317 | non-muscle invasive bladder cancer | Phase III; N=1055 | sasanlimab plus BCG induction with or without maintenance | BCG induction plus maintenance | Primary efficacy: 40450141 (2025) | BCG is an active immunotherapy comparator in both randomized groups. |
| CheckMate 238 | Expanded sensitivity | NCT02388906 | melanoma; stage IIIB,IIIC, or IV | Phase III; N=906 | nivolumab | ipilimumab | Primary efficacy: 28891423 (2017); Four-year efficacy: 32961119 (2020) | Ipilimumab is an active immunotherapy comparator. |
| SWOG S1404 | Expanded sensitivity | NCT02506153 | melanoma | Phase III; N=1303 | pembrolizumab | interferon alfa-2b or ipilimumab | Primary efficacy: 34764195 (2022) | Ipilimumab/high-dose interferon is an active control; comparator differs from placebo/observation. |
| E1609 | Expanded sensitivity | NCT01274338 | Melanoma; stage Ⅲ | Phase III; N=1670 | ipilimumab 3 mg/kg or 10 mg/kg | high-dose interferon alfa-2b | Primary efficacy: 31880964 (2020) | High-dose interferon is an active immunotherapy comparator. |
| NUTMEG | Expanded sensitivity | NCT04195139 | glioblastoma | Phase II; N=103 | nivolumab and temozolomide | temozolomide | Primary efficacy: PMC10576515 (2023) | Glioblastoma regimen after chemoradiotherapy; not a conventional adjuvant-only setting. |
| Toripalimab vs HDI | Expanded sensitivity | NCT03178123 | melanoma | Phase II; N=145 | toripalimab | high-dose interferon alfa-2b | Primary efficacy: 35842199 (2022) | High-dose interferon is an active immunotherapy comparator. |
| POTOMAC | Expanded sensitivity | NCT03528694 | non-muscle-invasive bladder cancer, NMIBC | Phase III; N=1018 | durvalumab plus BCG induction with or without maintenance | BCG induction plus maintenance | Primary efficacy: 41115436 (2025) | BCG is an active immunotherapy comparator in both randomized groups. |
| CheckMate 915 | Expanded sensitivity | NCT03068455 | Melanoma; stage IIIB-D or IV | Phase III; N=1844 | Nivolumab Combined With Ipilimumab | Nivolumab Alone | Primary efficacy: 36162037 (2023) | Nivolumab is an active immunotherapy comparator. |
| CIK HCC | Expanded sensitivity | NCT00699816 | hepatocellular carcinoma | Phase III; N=230 | autologous cytokine-induced killer cells | no adjuvant treatment | Primary efficacy: 25747273 (2015) | Primary paper was published in 2015, before the protocol publication window. |
| DIPPER | Expanded sensitivity | NCT03427827 | nasopharyngeal carcinoma | Phase III; N=450 | Camrelizumab | standard therapy/observation after chemoradiotherapy | None identified in the strict peer-reviewed dataset. | Post-chemoradiotherapy nasopharyngeal carcinoma setting rather than surgery-based adjuvant therapy. |
| 131I-metuximab HCC | Broad taxonomy sensitivity | NCT00819650 | hepatocellular carcinoma (HCC) | Phase II; N=156 | ¹³¹I-metuximab | no adjuvant treatment | Primary efficacy: 32164877 (2020) | Radioimmunotherapy/targeted-radioisotope mechanism is outside the core immunotherapy taxonomy. |

These 13 trials are retained to make protocol deviations and intervention-taxonomy decisions visible. The 12 eligibility-sensitivity trials remain outside the strict-primary main analysis and may contribute only to supplementary sensitivity analyses when a valid pair is available. The broad-taxonomy trial does not enter any quantitative model.

**Part C. Strict-set endpoint definitions, selected estimates, follow-up, and model status**

| **Trial/registration** | **Selected early endpoint** | **Trial-specific early-endpoint definition** | **Trial-specific OS definition** | **Selected treatment effects** | **Cutoff/follow-up** | **Exact estimate source** | **Model status/limitation** |
| --- | --- | --- | --- | --- | --- | --- | --- |
| IMpower010 NCT02486718 | DFS: Investigator-assessed DFS | Disease-Free Survival (DFS) in Intent-to-treat (ITT) Population: DFS was defined as the time from randomization to the first recurrence of NSCLC or occurrence of new primary NSCLC as determined by the investigator or death,… [Registry time frame: Up to 95 months] | Overall Survival (OS) in the ITT Population: OS was defined as the time from randomization to death from any cause. [Registry time frame: Baseline up to death from any cause (up to approximately 126 months)] | Early: HR 0.85 (95% CI 0.71–1.01); OS: HR 0.97 (95% CI 0.78–1.22) | 2024-01-26 cutoff; ≥5-year follow-up | PMID 40446184 | Include: DFS–OS primary. Second OS interim analysis |
| ADMEC-O NCT02196961 | DFS: Investigator-assessed DFS | Disease-free survival (DFS) rate at 12 months: The number of patients alive and free of disease at 12 months after randomization in arm A compared to DFS in arm B. [Registry time frame: 1 years post last patient first treatment/randomization] | Overall survival rate at 12 months: Overall survival rate at 12 months, defined as the number of patients alive at 12 months after randomization divided by the total number of patients randomized. [Registry time frame: 1 year post last patient first treatment/randomization] | Early: HR 0.58 (95% CI 0.3–1.12); OS: Not reported | Median follow-up 24.3 months | PMID 37451295 | Exclude from paired model. Only 16 deaths; no published OS HR |
| EORTC 18071 NCT00636168 | RFS: Recurrence-free survival | Recurrence Free Survival (RFS) Per Independent Review Committee…: Recurrence free survival (RFS) was programmatically determined based on the disease recurrence data provided by the IRC and was defined as the time between the date… [Registry time frame: Date of randomization to first date of recurrence or death or last available disease…] | Overall Survival in the Intent to Treat (ITT) Population: OS was defined as the time from the date of randomization to the date of death. For those participants who had not died, OS was censored at the recorded last… [Registry time frame: From June 2008 to January 2016 (approximately 90 months)] | Early: HR 0.76 (95% CI 0.64–0.89); OS: HR 0.72 (95.1% CI 0.58–0.88) | Median follow-up 5.3 years | PMID 27717298 | Include: RFS–OS primary. Endpoint-specific adjusted CI levels |
| CheckMate 274 NCT02632409 | DFS: Disease-free survival | Disease Free Survival (DFS): The time between the date of randomization and the date of first documented recurrence (local urothelial tract, local non-urothelial tract or distant), or death due… [Registry time frame: approximately up to 48 months] | Overall Survival: the time from randomization to the date of death from any cause. [Registry time frame: on going] | Early: HR 0.71 (95% CI 0.58–0.86); OS: HR 0.76 (95% CI 0.61–0.96) | Median follow-up 36.1 months | PMID 39393026 | Include: DFS–OS primary. Interim OS |
| KEYNOTE-564 NCT03142334 | DFS: Investigator-assessed DFS | Disease-free Survival (DFS) as Assessed by the Investigator: DFS, as assessed by the investigator, is defined as the time from randomization to the first documented local recurrence, distant kidney cancer metastasis(es), or… [Registry time frame: Up to approximately 42 months (database cutoff date 14 Dec 2020)] | Overall Survival (OS): OS was defined as the time from randomization to death due to any cause. [Registry time frame: Up to approximately 72 months] | Early: HR 0.72 (95% CI 0.59–0.87); OS: HR 0.62 (95% CI 0.44–0.87) | Median follow-up 57.2 months | PMID 38631003 | Include: DFS–OS primary. Third prespecified OS interim analysis |
| IMvigor010 NCT02450331 | DFS: Investigator-assessed DFS | Disease-Free Survival (DFS), as Assessed by Investigator: DFS is defined as the time from randomization to the time of first occurrence of a DFS event. DFS events include: local (pelvic) recurrence of UC (including soft… [Registry time frame: Randomization up to first occurrence of DFS event (up to approximately 50 months)] | Overall Survival (OS): Overall survival is defined as the time from randomization to the date of death from any cause, regardless of whether the death occurs during study treatment or… [Registry time frame: Randomization until death due to any cause (up to approximately 80 months)] | Early: HR 0.89 (95% CI 0.74–1.08); OS: HR 0.85 (95% CI 0.66–1.09) | Median follow-up 21.9 months | PMID 33721560 | Include: DFS–OS primary. First interim OS; hierarchical formal testing not permitted |
| PEARLS/KEYNOTE-091 NCT02504372 | DFS: Disease-free survival | Disease-Free Survival (DFS): DFS was defined as the time from randomization to either the date of disease recurrence or death (whatever the cause) as assessed by the investigator. Recurrence of… [Registry time frame: Up to approximately 84 months] | Overall Survival (OS): OS was defined as the time from randomization to the date of death. [Registry time frame: Up to approximately 132 months] | Early: HR 0.76 (95% CI 0.63–0.91); OS: HR 0.87 (95% CI 0.67–1.15) | 2021-09-20 cutoff; median follow-up 35.6 months | PMID 36108662 | Include: DFS–OS primary. OS approximately 18% mature |
| IMmotion010 NCT03024996 | DFS: Investigator-assessed DFS | Investigator-assessed Disease-Free Survival (DFS): Investigator-assessed DFS, defined as the time from randomization to death from any cause or the first documented recurrence assessed by investigator, whichever… [Registry time frame: From baseline up to first occurence of event by investigator assessment (up to…] | Overall Survival (OS): OS was defined as the time from randomization to death from any cause. [Registry time frame: From baseline up to death due to any cause (up to approximately 64 months)] | Early: HR 0.93 (95% CI 0.75–1.15); OS: HR 0.97 (95% CI 0.67–1.42) | 2022-05-03 cutoff; median follow-up 44.7 months | PMID 36099926 | Include: DFS–OS primary. OS approximately 14% mature |
| IMbrave050 NCT04102098 | RFS: IRF-assessed recurrence-free survival | Recurrence-Free Survival (RFS), as Determined by IRF: RFS is defined as the time from randomization to the first documented occurrence of intrahepatic or extrahepatic HCC as determined by an IRF, or death from any cause… [Registry time frame: Baseline up to approximately 33 months] | Overall Survival (OS): OS is defined as the time from randomization to death from any cause. [Registry time frame: Baseline up to approximately 91 months] | Early: HR 0.72 (95% CI 0.53–0.98); OS: HR 1.42 (95% CI 0.8–2.54) | 2022-10-21 cutoff; median follow-up 17.4 months | PMID 37871608 | Include: RFS–OS primary. OS highly immature; 47 deaths (7%) |
| C-POST NCT03969004 | DFS: Disease-free survival | DFS defined as time from randomization to the first documented…: For patients who do not have a tumor recurrence or death, DFS will be censored on the date of last disease assessment. [Registry time frame: Up to 54 months] | Overall survival (OS), defined as time from randomization to the… [Registry time frame: Up to 78 months] | Early: HR 0.32 (95% CI 0.2–0.51); OS: HR 0.86 (95% CI 0.39–1.9) | Primary DFS cutoff; median follow-up 24 months; 25 deaths | PMID 40454639; NEJM 2025 Supplementary Table S4 and Figure S5 | Include: DFS–OS primary. Immature OS: 12 vs 13 deaths (25 total); HR 0.86 (95% CI 0.39–1.90); placebo recipients could receive cemiplimab after recurrence |
| AMBASSADOR NCT03244384 | DFS: Disease-free survival | Disease-free survival: The stratified proportional hazards model will be the primary analysis. The hazard ratio and its 95% confidence interval from the stratified Cox model with a single… [Registry time frame: From randomization to the first metastatic recurrence (presence of any recurrent disease),…] | Overall survival: The stratified proportional hazards model will be the primary analysis. The hazard ratio and its 95% confidence interval from the stratified Cox model with a single… [Registry time frame: From randomization to the date of death from any cause, assessed up to 5 years] | Early: HR 0.73 (95% CI 0.59–0.9); OS: HR 0.98 (95% CI 0.76–1.26) | 2024-07-05 cutoff; median DFS follow-up 44.8 months | PMID 39282902 | Include: DFS–OS primary. OS interim analysis |
| Sintilimab HCC ChiCTR2000037655 | RFS: Recurrence-free survival | Not available in the machine-readable registry extract. | Not available in the machine-readable registry extract. | Early: HR 0.534 (95% CI 0.36–0.792); OS: HR 0.505 (95% CI 0.254–1.006) | Median follow-up 23.3 months | PMID 38242982 | Include: RFS–OS primary. OS immature; further follow-up requested by authors |
| ACT IV NCT01480479 | PFS: Progression-free survival in the MRD population | Progression-free survival: Compare progression-free survival between the two treatment arms [Registry time frame: Every 12 weeks from Day 1 through progression or initiation of other anti-cancer therapy] | Overall Survival: The primary efficacy endpoint of Overall Survival is defined as the number of months from randomization to the date of death (whatever the cause), and will be… [Registry time frame: During treatment and every three months from end of treatment through end of study or…] | Early: HR 1.01 (95% CI 0.8–1.29); OS: HR 1.01 (95% CI 0.79–1.3) | Final analysis after futility stop | PMID 28844499 | Descriptive only: PFS–OS. Only one strict PFS–OS trial |
| MAGRIT NCT00480025 | DFS: Disease-free survival | Person Year Rate (PYAR) as Regards Disease-free Survival (DFS) in…: DFS = time interval from randomization to 1st evidence of recurrence/death, if occurring before. All recurrence types were included, including local, regional \&… [Registry time frame: From administration of first dose of GSK1572932 study product/placebo solution to data lock…] | Person Year Rate (PYAR) as Regards Overall-survival (OS) in the…: OS was defined as the time interval from randomization to the date of death, irrespective of the cause of death. Patients still alive were censored at the last visit… [Registry time frame: From administration of first dose of GSK1572932 study product/placebo solution to data lock…] | Early: HR 1.02 (95% CI 0.89–1.18); OS: HR 1.04 (95% CI 0.86–1.24) | Median follow-up 38.1–39.5 months | PMID 27132212 | Include: DFS–OS primary. Analysis population excludes 40 randomized patients never treated |
| Seviprotimut-L NCT01546571 | RFS: Recurrence-free survival | Part B1: Recurrence Free Survival (RFS): This is an event driven trial. Recurrence-free survival time (RFS) is computed from the earliest of the date of recurrence or death or, if without recurrence or… [Registry time frame: 432 events or 8 years and 10 months] | Part B1: Overall Survival (OS): Overall survival (OS) analyses were based on the ITT analysis set; OS was defined as the duration from randomization until death. If the patient was alive, OS was… [Registry time frame: Overall survival (OS) was measured from randomization until death from any cause, assessed…] | Early: HR 0.881 (95% CI 0.629–1.233); OS: HR 0.64 (95% CI 0.34–1.18) | Part B1; up to 76 months | PMID 34599031 | Include: RFS–OS primary. Only 41 deaths |
| Adjuvant durvalumab ESCC NCT02520453 | DFS: Disease-free survival | Disease-free survival [Registry time frame: 1 year] | Not available in the machine-readable registry extract. | Early: HR 1.18 (95% CI 0.62–2.27); OS: HR 1.08 (95% CI 0.52–2.24) | Median follow-up 38.7 months | PMID 35158205 | Include: DFS–OS primary. Small phase II trial |
| IMMUNED NCT02523313 | RFS: Recurrence-free survival; nivolumab plus ipilimumab vs placebo | Efficacy of adjuvant immunotherapy with Nivolumab alone or in…: Recurrence-free survival (RFS) defined as the time from date of randomization until the date of the first recurrence (local or distant metastasis), new primary… [Registry time frame: 24 months after the last patient ended treatment] | Overall survival (OS): The OS of a patient is defined as the time from date of randomization until date of death. [Registry time frame: 24 months after the last patient ended treatment] | Early: HR 0.25 (97.5% CI 0.13–0.48); OS: HR 0.41 (95% CI 0.17–0.99) | Median follow-up 49.2 months | PMID 36099927 | Include: RFS–OS primary. Three-arm trial; one prespecified contrast selected |
| ATTRACTION-5 NCT03006705 | RFS: Central-assessed relapse-free survival | Relapse-free survival (RFS) [Registry time frame: 5 years] | Overall survival (OS) [Registry time frame: 5 years] | Early: HR 0.9 (95.72% CI 0.69–1.18); OS: HR 0.88 (95% CI 0.66–1.17) | Median time from first dose to cutoff 49.1 months | PMID 38906161 | Include: RFS–OS primary. Primary endpoint CI reported at 95.72% |
| CheckMate 914 Part A NCT03138512 | DFS: BICR-assessed disease-free survival | Disease-Free Survival (DFS) by BICR - Treatment Part A and B: Disease-Free Survival (DFS) is defined as the time from randomization to development of local disease recurrence (ie, recurrence of primary tumor in situ or… [Registry time frame: From randomization to development of local disease recurrence, distance metastasis, or…] | Overall Survival (OS) - Treatment Part A and B: Overall Survival (OS) is defined as the time between the date of randomization and the date of death. For participants without documentation of death, OS will be… [Registry time frame: From randomization to the date of death (up to approximately 72 months)] | Early: HR 0.92 (95% CI 0.71–1.19); OS: Not reported | Median follow-up 37.0 months | PMID 36774933 | Exclude from paired model. Only 61 deaths; no formal OS HR reported |
| MIND-DC NCT02993315 | RFS: Recurrence-free survival | Recurrence-free survival rate: The primary objective of this study is to determine whether adjuvant nDC vaccination, after complete radical lymph node dissection or sentinel node procedure in stage… [Registry time frame: 2 years] | Overall survival [Registry time frame: 2-years and median] | Early: HR 1.25 (90% CI 0.88–1.79); OS: HR 1.32 (90% CI 0.73–2.38) | Primary publication cutoff | PMID 38395969 | Include: RFS–OS primary. Both intervals are 90% CIs |
| CANOPY-A NCT03447769 | DFS: Investigator-assessed disease-free survival | Disease Free Survival (DFS) by Local Investigator: DFS is the time from the date of randomization to the date of the first documented NSCLC disease recurrence as assessed by local investigator radiologically or death… [Registry time frame: Up to approximately 4 years] | Overall Survival (OS): Overall Survival (OS) is the time from the date of randomization to the date of death due to any cause. The OS was censored at the latest date the subject was known… [Registry time frame: Up to approximately 4.3 years] | Early: HR 0.94 (95% CI 0.78–1.14); OS: HR 0.72 (95% CI 0.48–1.08) | Primary publication cutoff | PMID 37788412 | Include: DFS–OS primary. OS not formally tested after nonsignificant DFS |
| EORTC 1325/KEYNOTE-054 NCT02362594 | RFS: Recurrence-free survival | Part 1: Percentage of Participants With Recurrence-Free Survival…: RFS was defined as the time between the date of randomization and the date of first melanoma recurrence (local, regional, distant metastasis) or death (whatever the… [Registry time frame: 6 months] | Overall Survival (OS) for All Participants: OS will be defined as the time from the date of randomization to the date of death, whatever the cause. The follow-up of participants still alive will be censored at… [Registry time frame: Up to approximately 11 years] | Early: HR 0.61 (95% CI 0.51–0.72); OS: Not reported | Median follow-up 4.9 years | PMID 38319852 | Exclude from paired model. No peer-reviewed OS HR at the selected cutoff |
| CheckMate 76K NCT04099251 | RFS: Investigator-assessed recurrence-free survival | Recurrence Free Survival (RFS): Recurrence Free Survival (RFS) is defined as the time between the date of randomization and the date of first recurrence (local, regional or distant metastasis), new… [Registry time frame: From randomization up to the date of first recurrence, new primary melanoma, or death…] | Overall Survival (OS): OS is defined as the time between the date of randomization and the date of death. For those without documentation of death, OS will be censored on the last date the… [Registry time frame: From randomization up to the date of death or the last date the participant was known to be…] | Early: HR 0.42 (95% CI 0.3–0.59); OS: Not reported | Minimum follow-up 7.8 months | PMID 37845511 | Exclude from paired model. No published OS HR |

DFS and RFS retain their trial-specific labels and are modeled separately in the main analysis. No strict-primary EFS pair was available; PFS/PFS2 is analyzed only in the supplementary material. Time-specific 2-, 3-, and 5-year HR/95% CI cells, their direct or reconstructed source method, and nested cohort membership are reported in the analysis ledger and Table S5. Definitions here are concise reproductions of the trial-specific registry wording; complete definitions, time frames, source URLs, extraction/reconstruction flags, and machine-readable analysis files are retained in the supplied Supplementary Data and Statistical Code archive.

**Part D. Endpoint and source traceability for 12 eligibility-sensitivity and one broad-taxonomy trial**

| **Trial/registration** | **Registered primary family** | **Early-endpoint definition** | **OS definition** | **Exact publication mapping** | **Eligibility/taxonomy limitation** | **Quantitative role** |
| --- | --- | --- | --- | --- | --- | --- |
| ADRIATIC NCT03703297 | PFS; OS | PFS: Durvalumab Versus Placebo: Progression-Free Survival (PFS)…: PFS per RECIST 1.1 assessed by BICR was defined as the time from the date of randomization until the date of objective disease progression (PD) or death (by any cause… [Registry time frame: Response evaluations performed every 8 weeks (q8w) ± 1 week up to 72 weeks, then every 12…] | Durvalumab Versus Placebo: Overall Survival (OS): OS was defined as the time from the date of randomization until death due to any cause. Median OS was calculated using Kaplan-Meier method and its CI using… [Registry time frame: From date of randomization until death due to any cause, up to DCO date 15 January 2024 (a…] | Primary efficacy: 39268857 (2024) | Nonoperative post-chemoradiotherapy consolidation setting. | Contributes to supplementary eligibility sensitivity: 2y=no, 3y=no, 5y=no. |
| Ipi-Glio ISRCTN84434175 | OS | : ISRCTN record requires manual endpoint transcription: Primary-paper endpoint wording will be used provisionally and explicitly labeled. | Not available in the machine-readable registry extract. | Primary efficacy: 40800123 (2025) | Glioblastoma treatment begins after surgery and chemoradiotherapy; not a conventional adjuvant-only setting. | Contributes to supplementary eligibility sensitivity: 2y=no, 3y=no, 5y=no. |
| CREST NCT04165317 | EFS | EFS: Cohort A: Event Free Survival (EFS) as Assessed by the…: EFS: time from randomization till recurrence of high-grade disease, progression of disease, persistence of carcinoma in situ (CIS), death due to any cause, whichever… [Registry time frame: From randomization (Day 1) to first documentation of high-grade disease, progression of…] | Cohort A: Overall Survival (OS) for Participants: Arm A Versus Arm C: Overall survival was defined as the time in months from the date of randomization to the date of death due to any cause. Participants last known to be alive will be… [Registry time frame: From randomization (Day 1) until date of death due to any cause or censoring date] | Primary efficacy: 40450141 (2025) | BCG is an active immunotherapy comparator in both randomized groups. | Contributes to supplementary eligibility sensitivity: 2y=yes, 3y=yes, 5y=no. |
| CheckMate 238 NCT02388906 | RFS | RFS: Recurrence-free Survival (RFS): RFS is defined as the time between the date of randomization and the date of first recurrence (local, regional or distant metastasis), new primary melanoma, or death… [Registry time frame: up to 36 months] | Overall Survival (OS): OS is defined as as the time between the date of randomization and the date of death. [Registry time frame: up to 106.6 months] | Primary efficacy: 28891423 (2017); Four-year efficacy: 32961119 (2020) | Ipilimumab is an active immunotherapy comparator. | Contributes to supplementary eligibility sensitivity: 2y=yes, 3y=yes, 5y=yes. |
| SWOG S1404 NCT02506153 | OS | RFS: Relapse-free Survival (RFS): Time from date of randomization to date of first documentation of relapse or death due to any cause. Patients last known to be alive and relapse-free are censored at… [Registry time frame: 5 years after last randomization] | Overall Survival (OS): Time from date of randomization to date of death due to any cause. Patients known to be alive are censored at date of last contact. The results were presented as… [Registry time frame: 5 years after last randomization] | Primary efficacy: 34764195 (2022) | Ipilimumab/high-dose interferon is an active control; comparator differs from placebo/observation. | Contributes to supplementary eligibility sensitivity: 2y=yes, 3y=no, 5y=no. |
| E1609 NCT01274338 | RFS; OS | RFS: Recurrence-free Survival (RFS; Arm B [HDI] vs. Arm C [LIP]): Recurrence-free survival is defined as the time from randomization to recurrence or death, whichever occurs first. The following criteria constitute the only… [Registry time frame: Assessed every 3 months for 2 years, then every 6 months for years 3-5 and then annually up…] | 5-year Overall Survival (OS) Rate (Arm B [HDI] vs. Arm C [LIP]): Overall survival is defined as the time from randomization to death or date last known alive. [Registry time frame: Assessed every 3 months for 2 years, then every 6 months for years 3-5] | Primary efficacy: 31880964 (2020) | High-dose interferon is an active immunotherapy comparator. | Contributes to supplementary eligibility sensitivity: 2y=yes, 3y=yes, 5y=yes. |
| NUTMEG NCT04195139 | OS | PFS: Progression Free Survival: Progression free survival (PFS) is defined as the interval from date of randomisation to the date of first evidence of disease progression or death from any cause,… [Registry time frame: 6 months post randomisation] | Overall survival outcomes: Overall survival is defined as the interval from the date of randomisation to date of death from any cause, or date of last known follow-up alive. This will be… [Registry time frame: 24 months post randomisation of first participant] | Primary efficacy: PMC10576515 (2023) | Glioblastoma regimen after chemoradiotherapy; not a conventional adjuvant-only setting. | Contributes to supplementary eligibility sensitivity: 2y=no, 3y=no, 5y=no. |
| Toripalimab vs HDI NCT03178123 | RFS | RFS: Recurrence-free survival rate: to evaluate the Recurrence free survival (RFS) of the patients with mucosal melanoma treated by JS001 and high-Dose interferon [Registry time frame: 5 years] | Overall survival (OS): to evaluate the Overall survival (OS) of the patients with mucosal melanoma treated by JS001 and high-Dose interferon [Registry time frame: 5 years] | Primary efficacy: 35842199 (2022) | High-dose interferon is an active immunotherapy comparator. | Contributes to supplementary eligibility sensitivity: 2y=no, 3y=no, 5y=no. |
| POTOMAC NCT03528694 | DFS | DFS: The efficacy of Durvalumab + BCG (induction plus maintenance)…: Disease-free survival using Investigator disease assessments. [Registry time frame: Up to 4 years] | To assess the efficacy of durvalumab + BCG (induction and…: Overall survival (OS) is defined as the time from the date of randomization until death due to any cause regardless of whether the patient withdraws from randomized… [Registry time frame: Up to 7 years] | Primary efficacy: 41115436 (2025) | BCG is an active immunotherapy comparator in both randomized groups. | Contributes to supplementary eligibility sensitivity: 2y=yes, 3y=yes, 5y=yes. |
| CheckMate 915 NCT03068455 | RFS | RFS: Recurrence-free Survival (RFS) - All Randomized Participants: RFS was defined as the time between the date of randomization and the date of first recurrence (local, regional or distant metastasis), new primary melanoma… [Registry time frame: From randomization to Primary Completion Date (up to approximately 3 years)] | Overall Survival (OS) - All Randomized Participants: OS is defined as the time between the date of randomization and the date of death. Median based on Kaplan-Meier Estimates. [Registry time frame: From randomization to date of death (up to approximately 45 months)] | Primary efficacy: 36162037 (2023) | Nivolumab is an active immunotherapy comparator. | Contributes to supplementary eligibility sensitivity: 2y=yes, 3y=no, 5y=no. |
| CIK HCC NCT00699816 | RFS | RFS: Recurrence Free Survival(RFS): RFS was measured from the date of randomization to the first recurrence or to death from any cause. [Registry time frame: Every 3months from the baseline for 24 months and then every 3-6 months until the data…] | Overall Survival(OS): Overall survival was measured from the date of randomization until death from any cause. [Registry time frame: Every 3months from the baseline for 24 months, and then every 3-6 months until the data…] | Primary efficacy: 25747273 (2015) | Primary paper was published in 2015, before the protocol publication window. | Contributes to supplementary eligibility sensitivity: 2y=yes, 3y=no, 5y=no. |
| DIPPER NCT03427827 | EFS | RFS: locoregional recurrence-free survival: calculated from date of randomisation to the first locoregional failure [Registry time frame: 3 years] | overall survival: calculated from date of randomisation to death [Registry time frame: 5 years] | None identified in the strict peer-reviewed dataset. | Post-chemoradiotherapy nasopharyngeal carcinoma setting rather than surgery-based adjuvant therapy. | Contributes to supplementary eligibility sensitivity: 2y=yes, 3y=yes, 5y=no. |
| 131I-metuximab HCC NCT00819650 | OS | No non-OS endpoint identified: Not available in the machine-readable registry extract. | Overall survival [Registry time frame: 2010] | Primary efficacy: 32164877 (2020) | Radioimmunotherapy/targeted-radioisotope mechanism is outside the core immunotherapy taxonomy. | Descriptive audit only; excluded from every quantitative model. |

Parts B1–D provide registration, intervention, comparator, endpoint definition, publication mapping, follow-up or registry time frame, and analysis status for every one of the 36 retained unique trials. The quantitative role column distinguishes trials contributing only to supplementary eligibility sensitivities from those retained solely for descriptive evidence completeness. No broad-taxonomy study enters a quantitative model.

**Table S3. Outcome-specific Cochrane RoB 2 assessments**

| **Trial** | **Registration** | **Outcome** | **Exact source** | **Randomization** | **Deviations from intended interventions** | **Missing outcome data** | **Outcome measurement** | **Selection of reported result** | **Overall bias** | **Outcome-specific rationale** |
| --- | --- | --- | --- | --- | --- | --- | --- | --- | --- | --- |
| IMpower010 | NCT02486718 | DFS | PMID 40446184 | Some concerns | Some concerns | Low risk | Low risk | Some concerns | Some concerns | Selected DFS estimate: Investigator-assessed DFS. Replaces the old mixture of subgroup/cutoff estimates with a same-population pair. Population/cutoff check: ITT for both endpoints. |
| IMpower010 | NCT02486718 | OS | PMID 40446184 | Some concerns | Some concerns | Low risk | Low risk | Some concerns | Some concerns | Death is objective and the OS estimate was paired to the same randomized population/cutoff. Source-specific limitation: Second OS interim analysis. OS maturity was handled as imprecision and in sensitivity analysis, not recoded as missing-outcome bias. |
| ADMEC-O | NCT02196961 | DFS | PMID 37451295 | Some concerns | Some concerns | Low risk | Some concerns | Low risk | Some concerns | Selected DFS estimate: Investigator-assessed DFS. Retained in the evidence set but cannot contribute to an early-endpoint–OS association. Population/cutoff check: ITT; OS not mature. |
| EORTC 18071 | NCT00636168 | RFS | PMID 27717298 | Low risk | Low risk | Low risk | Low risk | Low risk | Low risk | Selected RFS estimate: Recurrence-free survival. Published same-cutoff RFS and OS pair; OS CI was reported at 95.1%. Population/cutoff check: ITT for both endpoints. |
| EORTC 18071 | NCT00636168 | OS | PMID 27717298 | Low risk | Low risk | Low risk | Low risk | Low risk | Low risk | Death is objective and the OS estimate was paired to the same randomized population/cutoff. Source-specific limitation: Endpoint-specific adjusted CI levels. OS maturity was handled as imprecision and in sensitivity analysis, not recoded as missing-outcome bias. |
| EORTC 18071 | NCT00636168 | QoL | PMID 28162999 | Low risk | Low risk | Some concerns | Some concerns | Some concerns | Some concerns | Repeated scheduled assessments; completion declined over time and multiple postbaseline time points were available. Source audit: Published table gives placebo minus ipilimumab +4.35 (2.07 to 6.64); harmonized here as treatment minus control. |
| CheckMate 274 | NCT02632409 | DFS | PMID 39393026 | Low risk | Low risk | Low risk | Low risk | Low risk | Low risk | Selected DFS estimate: Disease-free survival. Corrects the old pairing of ITT DFS with PD-L1-positive OS. Population/cutoff check: ITT for both endpoints. |
| CheckMate 274 | NCT02632409 | OS | PMID 39393026 | Low risk | Low risk | Low risk | Low risk | Low risk | Low risk | Death is objective and the OS estimate was paired to the same randomized population/cutoff. Source-specific limitation: Interim OS. OS maturity was handled as imprecision and in sensitivity analysis, not recoded as missing-outcome bias. |
| CheckMate 274 | NCT02632409 | QoL | PMID 35288066 | Low risk | Low risk | Some concerns | Some concerns | Some concerns | Some concerns | MMRM depends on assumptions about missing postbaseline observations; the reported estimand spans treatment and follow-up visits. Source audit: Old point estimate fell outside its CI. Correct source value is 0.10 (−2.00 to 2.20). Available-data rate declined to 38.5% vs 36.2% by week 49. |
| KEYNOTE-564 | NCT03142334 | DFS | PMID 38631003 | Some concerns | Some concerns | Some concerns | Some concerns | Some concerns | Some concerns | Selected DFS estimate: Investigator-assessed DFS. Replaces the old East-Asian subgroup estimates with the full randomized population. Population/cutoff check: ITT for both endpoints. |
| KEYNOTE-564 | NCT03142334 | OS | PMID 38631003 | Some concerns | Some concerns | Low risk | Low risk | Some concerns | Some concerns | Death is objective and the OS estimate was paired to the same randomized population/cutoff. Source-specific limitation: Third prespecified OS interim analysis. OS maturity was handled as imprecision and in sensitivity analysis, not recoded as missing-outcome bias. |
| KEYNOTE-564 | NCT03142334 | QoL | PMID 37589219 | Some concerns | Some concerns | Some concerns | Some concerns | Some concerns | Some concerns | Week-52 change uses an available longitudinal analysis population and is sensitive to missing postbaseline data. Source audit: Required QoL publication was omitted from the old QoL sheet. Completion fell from about 92–94% at baseline to about 63–66% at week 52. |
| IMvigor010 | NCT02450331 | DFS | PMID 33721560 | Low risk | Low risk | Low risk | Low risk | Low risk | Low risk | Selected DFS estimate: Investigator-assessed DFS. Uses the primary-paper same-cutoff pair rather than mixing later registry OS with the earlier DFS analysis. Population/cutoff check: ITT for both endpoints. |
| IMvigor010 | NCT02450331 | OS | PMID 33721560 | Low risk | Low risk | Low risk | Low risk | Low risk | Low risk | Death is objective and the OS estimate was paired to the same randomized population/cutoff. Source-specific limitation: First interim OS; hierarchical formal testing not permitted. OS maturity was handled as imprecision and in sensitivity analysis, not recoded as missing-outcome bias. |
| PEARLS/KEYNOTE-091 | NCT02504372 | DFS | PMID 36108662 | Low risk | Low risk | Low risk | Low risk | Low risk | Low risk | Selected DFS estimate: Disease-free survival. Same randomized population and cutoff; immaturity is retained for sensitivity analysis. Population/cutoff check: ITT for both endpoints. |
| PEARLS/KEYNOTE-091 | NCT02504372 | OS | PMID 36108662 | Low risk | Low risk | Low risk | Low risk | Low risk | Low risk | Death is objective and the OS estimate was paired to the same randomized population/cutoff. Source-specific limitation: OS approximately 18% mature. OS maturity was handled as imprecision and in sensitivity analysis, not recoded as missing-outcome bias. |
| IMmotion010 | NCT03024996 | DFS | PMID 36099926 | Some concerns | Some concerns | Low risk | Some concerns | Some concerns | Some concerns | Selected DFS estimate: Investigator-assessed DFS. Published same-cutoff pair; flagged for an OS-maturity sensitivity analysis. Population/cutoff check: ITT for both endpoints. |
| IMmotion010 | NCT03024996 | OS | PMID 36099926 | Some concerns | Some concerns | Low risk | Low risk | Some concerns | Some concerns | Death is objective and the OS estimate was paired to the same randomized population/cutoff. Source-specific limitation: OS approximately 14% mature. OS maturity was handled as imprecision and in sensitivity analysis, not recoded as missing-outcome bias. |
| IMbrave050 | NCT04102098 | RFS | PMID 37871608 | Low risk | Some concerns | Low risk | Some concerns | Some concerns | Some concerns | Selected RFS estimate: IRF-assessed recurrence-free survival. The same-cutoff OS estimate is retained but will be removed in the maturity sensitivity analysis. Population/cutoff check: ITT for both endpoints. |
| IMbrave050 | NCT04102098 | OS | PMID 37871608 | Low risk | Some concerns | Low risk | Low risk | Some concerns | Some concerns | Death is objective and the OS estimate was paired to the same randomized population/cutoff. Source-specific limitation: OS highly immature; 47 deaths (7%). OS maturity was handled as imprecision and in sensitivity analysis, not recoded as missing-outcome bias. |
| C-POST | NCT03969004 | DFS | PMID 40454639; NEJM 2025 Supplementary Table S4 and Figure S5 | Low risk | Low risk | Low risk | Low risk | Low risk | Low risk | Selected DFS estimate: Disease-free survival. The previously historical OS HR is directly reported in peer-reviewed Supplementary Table S4 and Figure S5. It is retained in the full paired analysis and excluded from the OS-maturity sensitivity analysis. Population/cutoff check: ITT; DFS and OS from the same randomized analysis and cutoff. |
| C-POST | NCT03969004 | OS | PMID 40454639; NEJM 2025 Supplementary Table S4 and Figure S5 | Low risk | Low risk | Low risk | Low risk | Low risk | Low risk | Death is objective and the OS estimate was paired to the same randomized population/cutoff. Source-specific limitation: Immature OS: 12 vs 13 deaths (25 total); HR 0.86 (95% CI 0.39–1.90); placebo recipients could receive cemiplimab after recurrence. OS maturity was handled as imprecision and in sensitivity analysis, not recoded as missing-outcome bias. |
| C-POST | NCT03969004 | QoL | PMID 40454639; Supplementary Table S12 | Low risk | Low risk | Some concerns | Some concerns | Some concerns | Some concerns | The overall treatment-period MMRM used 164 cemiplimab and 163 placebo participants, fewer than the 209 and 206 randomized; observed visit-level availability declined during follow-up. Source audit: The peer-reviewed NEJM primary article and Supplementary Table S12 report an overall treatment-minus-placebo least-squares mean difference of -0.94 points (95% CI -3.65 to 1.77; n=164 vs 163). Figure S6 was independently calibrated in WebPlotDigitizer to document the observed mean trajectories; the exact table estimate is used for modeling. |
| AMBASSADOR | NCT03244384 | DFS | PMID 39282902 | Low risk | Low risk | Low risk | Low risk | Low risk | Low risk | Selected DFS estimate: Disease-free survival. Same randomized population and cutoff in the primary report. Population/cutoff check: ITT for both endpoints. |
| AMBASSADOR | NCT03244384 | OS | PMID 39282902 | Low risk | Low risk | Low risk | Low risk | Low risk | Low risk | Death is objective and the OS estimate was paired to the same randomized population/cutoff. Source-specific limitation: OS interim analysis. OS maturity was handled as imprecision and in sensitivity analysis, not recoded as missing-outcome bias. |
| Sintilimab HCC | ChiCTR2000037655 | RFS | PMID 38242982 | Some concerns | Some concerns | Some concerns | Some concerns | Some concerns | Some concerns | Selected RFS estimate: Recurrence-free survival. The OS estimate is source-reported but is flagged for the maturity sensitivity analysis. Population/cutoff check: ITT for both endpoints. |
| Sintilimab HCC | ChiCTR2000037655 | OS | PMID 38242982 | Some concerns | Some concerns | Low risk | Low risk | Some concerns | Some concerns | Death is objective and the OS estimate was paired to the same randomized population/cutoff. Source-specific limitation: OS immature; further follow-up requested by authors. OS maturity was handled as imprecision and in sensitivity analysis, not recoded as missing-outcome bias. |
| ACT IV | NCT01480479 | PFS | PMID 28844499 | Low risk | Low risk | Low risk | Low risk | Low risk | Low risk | Selected PFS estimate: Progression-free survival in the MRD population. Not combined with DFS or RFS because the endpoint definition and disease setting are distinct. Population/cutoff check: Minimal-residual-disease population for both endpoints. |
| ACT IV | NCT01480479 | OS | PMID 28844499 | Low risk | Low risk | Low risk | Low risk | Low risk | Low risk | Death is objective and the OS estimate was paired to the same randomized population/cutoff. Source-specific limitation: Only one strict PFS–OS trial. OS maturity was handled as imprecision and in sensitivity analysis, not recoded as missing-outcome bias. |
| MAGRIT | NCT00480025 | DFS | PMID 27132212 | Low risk | Low risk | Low risk | Low risk | Low risk | Low risk | Selected DFS estimate: Disease-free survival. The source-defined efficacy population is retained and labeled rather than silently treated as ITT. Population/cutoff check: Total treated randomized population for both endpoints. |
| MAGRIT | NCT00480025 | OS | PMID 27132212 | Low risk | Low risk | Low risk | Low risk | Low risk | Low risk | Death is objective and the OS estimate was paired to the same randomized population/cutoff. Source-specific limitation: Analysis population excludes 40 randomized patients never treated. OS maturity was handled as imprecision and in sensitivity analysis, not recoded as missing-outcome bias. |
| Seviprotimut-L | NCT01546571 | RFS | PMID 34599031 | Low risk | Low risk | Low risk | Low risk | Low risk | Low risk | Selected RFS estimate: Recurrence-free survival. Published OS secondary endpoint and same randomized comparison. Population/cutoff check: ITT Part B1 for both endpoints. |
| Seviprotimut-L | NCT01546571 | OS | PMID 34599031 | Low risk | Low risk | Low risk | Low risk | Low risk | Low risk | Death is objective and the OS estimate was paired to the same randomized population/cutoff. Source-specific limitation: Only 41 deaths. OS maturity was handled as imprecision and in sensitivity analysis, not recoded as missing-outcome bias. |
| Adjuvant durvalumab ESCC | NCT02520453 | DFS | PMID 35158205 | Low risk | Some concerns | Low risk | Low risk | Low risk | Some concerns | Selected DFS estimate: Disease-free survival. Same-cutoff randomized DFS and OS pair. Population/cutoff check: ITT for both endpoints. |
| Adjuvant durvalumab ESCC | NCT02520453 | OS | PMID 35158205 | Low risk | Some concerns | Low risk | Low risk | Low risk | Some concerns | Death is objective and the OS estimate was paired to the same randomized population/cutoff. Source-specific limitation: Small phase II trial. OS maturity was handled as imprecision and in sensitivity analysis, not recoded as missing-outcome bias. |
| IMMUNED | NCT02523313 | RFS | PMID 36099927 | Low risk | Low risk | Low risk | Low risk | Low risk | Low risk | Selected RFS estimate: Recurrence-free survival; nivolumab plus ipilimumab vs placebo. The endpoint-specific CI levels and selected contrast are explicit. Population/cutoff check: ITT; combination arm vs placebo for both endpoints. |
| IMMUNED | NCT02523313 | OS | PMID 36099927 | Low risk | Low risk | Low risk | Low risk | Low risk | Low risk | Death is objective and the OS estimate was paired to the same randomized population/cutoff. Source-specific limitation: Three-arm trial; one prespecified contrast selected. OS maturity was handled as imprecision and in sensitivity analysis, not recoded as missing-outcome bias. |
| ATTRACTION-5 | NCT03006705 | RFS | PMID 38906161 | Low risk | Low risk | Low risk | Low risk | Low risk | Low risk | Selected RFS estimate: Central-assessed relapse-free survival. Same randomized population and cutoff; exact nonstandard RFS CI level is retained. Population/cutoff check: ITT for both endpoints. |
| ATTRACTION-5 | NCT03006705 | OS | PMID 38906161 | Low risk | Low risk | Low risk | Low risk | Low risk | Low risk | Death is objective and the OS estimate was paired to the same randomized population/cutoff. Source-specific limitation: Primary endpoint CI reported at 95.72%. OS maturity was handled as imprecision and in sensitivity analysis, not recoded as missing-outcome bias. |
| CheckMate 914 Part A | NCT03138512 | DFS | PMID 36774933 | Low risk | Low risk | Low risk | Low risk | Low risk | Low risk | Selected DFS estimate: BICR-assessed disease-free survival. The old OS value 0.62 (0.15–2.60) is not used because its source and estimand were not reproducibly documented. Population/cutoff check: ITT; planned OS interim threshold not reached. |
| MIND-DC | NCT02993315 | RFS | PMID 38395969 | Low risk | Low risk | Low risk | Low risk | Low risk | Low risk | Selected RFS estimate: Recurrence-free survival. The CI level is converted correctly when deriving standard errors. Population/cutoff check: ITT for both endpoints. |
| MIND-DC | NCT02993315 | OS | PMID 38395969 | Low risk | Low risk | Low risk | Low risk | Low risk | Low risk | Death is objective and the OS estimate was paired to the same randomized population/cutoff. Source-specific limitation: Both intervals are 90% CIs. OS maturity was handled as imprecision and in sensitivity analysis, not recoded as missing-outcome bias. |
| CANOPY-A | NCT03447769 | DFS | PMID 37788412 | Low risk | Low risk | Low risk | Low risk | Low risk | Low risk | Selected DFS estimate: Investigator-assessed disease-free survival. The source-reported descriptive OS estimate is retained and flagged for sensitivity analysis. Population/cutoff check: Full analysis set for both endpoints. |
| CANOPY-A | NCT03447769 | OS | PMID 37788412 | Low risk | Low risk | Low risk | Low risk | Low risk | Low risk | Death is objective and the OS estimate was paired to the same randomized population/cutoff. Source-specific limitation: OS not formally tested after nonsignificant DFS. OS maturity was handled as imprecision and in sensitivity analysis, not recoded as missing-outcome bias. |
| EORTC 1325/KEYNOTE-054 | NCT02362594 | RFS | PMID 38319852 | Low risk | Low risk | Low risk | Low risk | Low risk | Low risk | Selected RFS estimate: Recurrence-free survival. Retained for efficacy and QoL evidence but cannot contribute to RFS–OS modeling. Population/cutoff check: ITT; OS not reported. |
| EORTC 1325/KEYNOTE-054 | NCT02362594 | QoL | PMID 39146951 | Low risk | Low risk | Some concerns | Some concerns | Some concerns | Some concerns | The years 2–4 average is conditional on long-term PRO availability and differs from early assessment windows in other trials. Source audit: Long-term assessment window; compliance 93% at baseline and 60–90% thereafter. |
| CheckMate 76K | NCT04099251 | RFS | PMID 37845511 | Low risk | Low risk | Low risk | Low risk | Low risk | Low risk | Selected RFS estimate: Investigator-assessed recurrence-free survival. Retained for efficacy and QoL evidence but cannot contribute to RFS–OS modeling. Population/cutoff check: ITT; OS not reported. |
| CheckMate 76K | NCT04099251 | QoL | PMID 40139004 | Low risk | Low risk | Some concerns | Some concerns | Some concerns | Some concerns | The estimate summarizes on-treatment visits with short follow-up and incomplete postbaseline availability. Source audit: Peer-reviewed dedicated PRO paper; randomized patients with at least one complete baseline or on-treatment PRO. |

RoB 2 was applied separately to the selected recurrence endpoint, OS, and QoL. No OS judgment is shown when no extractable OS HR was reported. OS maturity was treated as a precision limitation. QoL judgments explicitly considered missing postbaseline questionnaires, analysis population, domain/time-point selection, and the participant-reported nature of measurement.

**Table S4. Verified and provisional global-health/QoL contrasts and prespecified model-availability rules**

**Part A. Trial-level global-health/QoL contrasts**

| **Trial** | **Registration** | **Analysis set** | **Treatment-control MD (95% CI)** | **QoL domain** | **Assessment/estimand** | **Source tier** | **Model set** | **Use decision** | **Audit finding** |
| --- | --- | --- | --- | --- | --- | --- | --- | --- | --- |
| C-POST | NCT03969004 | Strict primary | -0.94 (-3.65 to 1.77) | Global health status / quality of life | Overall treatment-period MMRM least-squares mean change from baseline; cemiplimab minus placebo | corrected exact contrast | Verified exact QoL | Include in strict exploratory QoL regression | The peer-reviewed NEJM primary article and Supplementary Table S12 report an overall treatment-minus-placebo least-squares mean difference of -0.94 points (95% CI -3.65 to 1.77; n=164 vs 163). Figure S6 was independently calibrated in WebPlotDigitizer to document the observed mean trajectories; the exact table estimate is used for modeling. |
| CheckMate 274 | NCT02632409 | Strict primary | 0.1 (-2 to 2.2) | Global health status/QoL | MMRM overall LS mean change across treatment and follow-up visits | corrected exact contrast | Verified exact QoL | Include in strict exploratory set | Old point estimate fell outside its CI. Correct source value is 0.10 (−2.00 to 2.20). Available-data rate declined to 38.5% vs 36.2% by week 49. |
| CheckMate 76K | NCT04099251 | Strict primary | -1.3 (-2.9 to 0.4) | Global health status/QoL | Overall MMRM LS mean difference across on-treatment visits | corrected exact contrast | Verified exact QoL | Include in strict exploratory set | Peer-reviewed dedicated PRO paper; randomized patients with at least one complete baseline or on-treatment PRO. |
| EORTC 1325/KEYNOTE-054 | NCT02362594 | Strict primary | -2.19 (-4.65 to 0.27) | Global QoL | Mean change from baseline to average QoL between years 2 and 4 | corrected exact contrast | Verified exact QoL | Include in strict exploratory set | Long-term assessment window; compliance 93% at baseline and 60–90% thereafter. |
| EORTC 18071 | NCT00636168 | Strict primary | -4.35 (-6.64 to -2.07) | Global health/QoL | Average during induction; ITT; repeated scheduled assessments | corrected exact contrast | Verified exact QoL | Include in strict exploratory set | Published table gives placebo minus ipilimumab +4.35 (2.07 to 6.64); harmonized here as treatment minus control. |
| KEYNOTE-564 | NCT03142334 | Strict primary | -2.5 (-5.2 to 0.1) | Global health status/QoL | Baseline-adjusted LS mean difference in change at week 52 | corrected exact contrast | Verified exact QoL | Include in strict exploratory set | Required QoL publication was omitted from the old QoL sheet. Completion fell from about 92–94% at baseline to about 63–66% at week 52. |
| CREST | NCT04165317 | Expanded sensitivity | -4.7 (-8.24 to -1.16) | Global health status / quality of life | End of treatment; unadjusted treatment-minus-control contrast derived from exact arm-level change means, SDs, and sample sizes | corrected exact contrast | Verified exact QoL | Expanded sensitivity only; exclude from strict QoL regression | Peer-reviewed Supplementary Table 3 reports end-of-treatment global-health changes of -5.7 (SD 21.27; n=249) with sasanlimab plus BCG-I+M and -1.0 (SD 19.68; n=269) with BCG-I+M. The derived treatment-minus-control mean difference is -4.70 (95% CI -8.24 to -1.16), which confirms the historical value and corrects the earlier conclusion that no peer-reviewed table supported it. WebPlotDigitizer was used only as a figure-level cross-check because exact table values are available. |
| SWOG S1404 | NCT02506153 | Expanded sensitivity | 6.7 (4.9 to 8.5) | Global health score | Baseline-adjusted cycle 3 contrast | corrected exact contrast | Verified exact QoL | Expanded sensitivity only | Old sign was reversed. Active-control trial; 832 patients evaluable for the primary QoL endpoint. |
| CheckMate 238 | NCT02388906 | Expanded sensitivity | -2.6 (-6.17 to 0.97) | Global health status/QoL (provisional) | Exact assessment window pending | historical provisional contrast | Expanded provisional QoL | Pending; expanded only | Active ipilimumab comparator; source-table verification required. |
| CheckMate 915 | NCT03068455 | Expanded sensitivity | -2.6 (-8.1 to 3) | Global health status/QoL (provisional) | Exact estimand/time aggregation pending source-table verification | historical provisional contrast | Expanded provisional QoL | Pending; expanded only | Active nivolumab comparator and source-location verification required. |
| DIPPER | NCT03427827 | Expanded sensitivity | -2.66 (-5.91 to 0.6) | Global health status/QoL (provisional) | Three-year repeated assessment; exact eTable estimand pending | historical provisional contrast | Expanded provisional QoL | Pending; expanded only | HRQoL population 166 vs 168; changes were reported as not significantly different over 3 years. |
| NUTMEG | NCT04195139 | Expanded sensitivity | -0.5 (-13.8 to 12.8) | Global health status/QoL | Repeated assessments during adjuvant therapy | historical provisional contrast | Expanded provisional QoL | Pending; expanded only | Completion declined from 91% at baseline to 50% at end of treatment; very imprecise estimate. |

**Part B. Time-specific model availability**

| **Role** | **QoL dataset** | **Survival comparison** | **Contrasts, n** | **Analysis status** | **Reason** |
| --- | --- | --- | --- | --- | --- |
| Primary QoL | Strict-primary verified exact QoL | 2y early vs 2y OS | 3 | Descriptive only | Descriptive only: fewer than 5 verified exact contrasts or no predictor variation |
| Primary QoL | Strict-primary verified exact QoL | 2y early vs 3y OS | 2 | Descriptive only | Descriptive only: fewer than 5 verified exact contrasts or no predictor variation |
| Primary QoL | Strict-primary verified exact QoL | 2y early vs 5y OS | 2 | Descriptive only | Descriptive only: fewer than 5 verified exact contrasts or no predictor variation |
| Primary QoL | Strict-primary verified exact QoL | 3y early vs 3y OS | 2 | Descriptive only | Descriptive only: fewer than 5 verified exact contrasts or no predictor variation |
| Primary QoL | Strict-primary verified exact QoL | 3y early vs 5y OS | 2 | Descriptive only | Descriptive only: fewer than 5 verified exact contrasts or no predictor variation |
| Primary QoL | Strict-primary verified exact QoL | 5y early vs 5y OS | 2 | Descriptive only | Descriptive only: fewer than 5 verified exact contrasts or no predictor variation |
| Supplementary sensitivity | Verified exact QoL including eligibility-sensitivity | 2y early vs 2y OS | 5 | Fitted | Verified-exact model fitted |
| Supplementary sensitivity | Verified exact QoL including eligibility-sensitivity | 2y early vs 3y OS | 3 | Descriptive only | Descriptive only: fewer than 5 verified exact contrasts or no predictor variation |
| Supplementary sensitivity | Verified exact QoL including eligibility-sensitivity | 2y early vs 5y OS | 2 | Descriptive only | Descriptive only: fewer than 5 verified exact contrasts or no predictor variation |
| Supplementary sensitivity | Verified exact QoL including eligibility-sensitivity | 3y early vs 3y OS | 3 | Descriptive only | Descriptive only: fewer than 5 verified exact contrasts or no predictor variation |
| Supplementary sensitivity | Verified exact QoL including eligibility-sensitivity | 3y early vs 5y OS | 2 | Descriptive only | Descriptive only: fewer than 5 verified exact contrasts or no predictor variation |
| Supplementary sensitivity | Verified exact QoL including eligibility-sensitivity | 5y early vs 5y OS | 2 | Descriptive only | Descriptive only: fewer than 5 verified exact contrasts or no predictor variation |

**Part C. Fitted REML models (verified exact contrasts and n≥5 only)**

| **QoL dataset** | **Role** | **Survival comparison** | **Predictor** | **Trials, n** | **REML slope (95% CI)** | **P value** | **Pseudo R²** | **I²** | **τ²** | **Interpretation** |
| --- | --- | --- | --- | --- | --- | --- | --- | --- | --- | --- |
| Verified exact QoL including eligibility-sensitivity | Supplementary sensitivity | 2y early vs 2y OS | Early endpoint HR | 5 | -1.304 (-15.273 to 12.664) | 0.855 | 0% | 95.1% | 26.804 | Supplementary eligibility sensitivity; no clear association. |
| Verified exact QoL including eligibility-sensitivity | Supplementary sensitivity | 2y early vs 2y OS | OS HR | 5 | -1.03 (-38.126 to 36.067) | 0.957 | 0% | 95.1% | 27.019 | Supplementary eligibility sensitivity; no clear association. |

QoL is treatment minus control, with higher global-health scores indicating better health. The primary QoL evidence is restricted to verified exact contrasts from strict-primary trials. Every strict time comparison had fewer than five contrasts, so no primary QoL slope, P value, or R² was estimated. The only fitted models used five verified exact contrasts after adding two eligibility-sensitivity trials and are therefore supplementary sensitivities. The four provisional contrasts are descriptive only and never enter a fitted model. Leave-one-out QoL slopes are not reported because each refit would have n=4.

**Table S5. Co-primary measurement-error analysis and supplementary robustness, eligibility, direct-HR, EFS, and PFS analyses**

**Part A. Strict-primary membership in the nested cohorts**

| **Trial** | **Registration** | **Analysis set** | **Early endpoint** | **2-year cohort** | **3-year cohort** | **5-year cohort** | **Nesting check** |
| --- | --- | --- | --- | --- | --- | --- | --- |
| IMpower010 | NCT02486718 | Strict primary | DFS | yes | yes | yes | pass |
| ADMEC-O | NCT02196961 | Strict primary | DFS | yes | no | no | pass |
| EORTC 18071 | NCT00636168 | Strict primary | RFS | yes | yes | yes | pass |
| CheckMate 274 | NCT02632409 | Strict primary | DFS | yes | yes | yes | pass |
| KEYNOTE-564 | NCT03142334 | Strict primary | DFS | no | no | no | pass |
| IMvigor010 | NCT02450331 | Strict primary | DFS | yes | no | no | pass |
| PEARLS/KEYNOTE-091 | NCT02504372 | Strict primary | DFS | yes | no | no | pass |
| IMmotion010 | NCT03024996 | Strict primary | DFS | yes | no | no | pass |
| IMbrave050 | NCT04102098 | Strict primary | RFS | yes | no | no | pass |
| C-POST | NCT03969004 | Strict primary | DFS | yes | no | no | pass |
| AMBASSADOR | NCT03244384 | Strict primary | DFS | yes | no | no | pass |
| Sintilimab HCC | ChiCTR2000037655 | Strict primary | RFS | yes | no | no | pass |
| ACT IV | NCT01480479 | Strict primary | PFS | no | no | no | pass |
| MAGRIT | NCT00480025 | Strict primary | DFS | yes | yes | yes | pass |
| Seviprotimut-L | NCT01546571 | Strict primary | RFS | yes | no | no | pass |
| Adjuvant durvalumab ESCC | NCT02520453 | Strict primary | DFS | yes | yes | no | pass |
| IMMUNED | NCT02523313 | Strict primary | RFS | yes | yes | no | pass |
| ATTRACTION-5 | NCT03006705 | Strict primary | RFS | yes | yes | yes | pass |
| CheckMate 914 Part A | NCT03138512 | Strict primary | DFS | yes | no | no | pass |
| MIND-DC | NCT02993315 | Strict primary | RFS | yes | yes | no | pass |
| CANOPY-A | NCT03447769 | Strict primary | DFS | yes | no | no | pass |
| EORTC 1325/KEYNOTE-054 | NCT02362594 | Strict primary | RFS | no | no | no | pass |
| CheckMate 76K | NCT04099251 | Strict primary | RFS | no | no | no | pass |

**Part B. Strict-primary bootstrap, cross-validation, and leave-one-out summaries**

| **Endpoint** | **Comparison** | **Trials, n** | **Slope (model 95% CI)** | **Bootstrap slope 95% CI** | **Weighted R² (bootstrap 95% CI)** | **Cross-validated R²** | **Leave-one-out slope range** |
| --- | --- | --- | --- | --- | --- | --- | --- |
| DFS | 2y early vs 2y OS | 12 | 0.014 (-0.286 to 0.314) | -0.326 to 0.287 | 0.001 (0 to 0.361) | -0.418 | -0.071 to 0.061 |
| DFS | 2y early vs 3y OS | 4 | 0.591 (-0.161 to 1.344) | 0.111 to 1.31 | 0.851 (0.492 to 1) | -0.045 | 0.267 to 0.805 |
| DFS | 2y early vs 5y OS | 3 | 0.681 (-1.737 to 3.098) | 0.393 to 1.14 | 0.927 (0.927 to 1) | — | Not estimated |
| DFS | 3y early vs 3y OS | 4 | 0.608 (-0.25 to 1.465) | 0.108 to 1.517 | 0.823 (0.43 to 1) | -0.269 | 0.253 to 0.876 |
| DFS | 3y early vs 5y OS | 3 | 0.695 (-2.194 to 3.584) | 0.382 to 1.319 | 0.903 (0.903 to 1) | — | Not estimated |
| DFS | 5y early vs 5y OS | 3 | 0.596 (-0.928 to 2.121) | 0.382 to 0.834 | 0.961 (0.961 to 1) | — | Not estimated |
| RFS | 2y early vs 2y OS | 7 | 0.63 (-1.243 to 2.502) | -0.966 to 1.689 | 0.13 (0 to 0.891) | -0.38 | 0.352 to 0.945 |
| RFS | 2y early vs 3y OS | 4 | 1.011 (0.416 to 1.607) | 0.779 to 1.435 | 0.964 (0.947 to 1) | 0.881 | 0.868 to 1.161 |
| RFS | 3y early vs 3y OS | 4 | 0.66 (-0.077 to 1.398) | 0.52 to 1.363 | 0.881 (0.873 to 1) | 0.436 | 0.536 to 1.015 |

Bootstrap results use 1,000 trial-level resamples. Leave-one-out models are not estimated when the full model contains only three trials. Negative cross-validated R² indicates that held-out predictions perform worse than the weighted mean and should not be interpreted as a negative biological association.

**Part C. Endpoint-specific eligibility-sensitivity analyses**

| **Endpoint** | **Comparison** | **Trials, n** | **Slope (95% CI)** | **P value** | **Weighted R²** | **Role** |
| --- | --- | --- | --- | --- | --- | --- |
| DFS | 2y early vs 2y OS | 13 | 0.016 (-0.265 to 0.297) | 0.904 | 0.001 | Supplementary eligibility sensitivity; broad taxonomy excluded. |
| DFS | 2y early vs 3y OS | 5 | 0.568 (0.079 to 1.057) | 0.034 | 0.82 | Supplementary eligibility sensitivity; broad taxonomy excluded. |
| DFS | 2y early vs 5y OS | 4 | 0.723 (0.055 to 1.392) | 0.043 | 0.916 | Supplementary eligibility sensitivity; broad taxonomy excluded. |
| DFS | 3y early vs 3y OS | 5 | 0.547 (-0.043 to 1.136) | 0.06 | 0.744 | Supplementary eligibility sensitivity; broad taxonomy excluded. |
| DFS | 3y early vs 5y OS | 4 | 0.688 (0.088 to 1.288) | 0.039 | 0.924 | Supplementary eligibility sensitivity; broad taxonomy excluded. |
| DFS | 5y early vs 5y OS | 4 | 0.617 (0.255 to 0.979) | 0.018 | 0.964 | Supplementary eligibility sensitivity; broad taxonomy excluded. |
| RFS | 2y early vs 2y OS | 12 | 0.83 (-0.505 to 2.165) | 0.196 | 0.161 | Supplementary eligibility sensitivity; broad taxonomy excluded. |
| RFS | 2y early vs 3y OS | 6 | 0.552 (-0.905 to 2.01) | 0.352 | 0.217 | Supplementary eligibility sensitivity; broad taxonomy excluded. |
| RFS | 2y early vs 5y OS | 4 | -0.028 (-3.341 to 3.286) | 0.975 | 0.001 | Supplementary eligibility sensitivity; broad taxonomy excluded. |
| RFS | 3y early vs 3y OS | 6 | 0.547 (-0.394 to 1.488) | 0.182 | 0.394 | Supplementary eligibility sensitivity; broad taxonomy excluded. |
| RFS | 3y early vs 5y OS | 4 | 0.436 (-3.161 to 4.033) | 0.654 | 0.12 | Supplementary eligibility sensitivity; broad taxonomy excluded. |
| RFS | 5y early vs 5y OS | 4 | 0.436 (-3.161 to 4.033) | 0.654 | 0.12 | Supplementary eligibility sensitivity; broad taxonomy excluded. |

**Part D. Directly reported HR-only sensitivity analyses**

| **Evidence set** | **Comparison** | **Trials, n** | **Slope (95% CI)** | **P value** | **Weighted R²** | **Source restriction** |
| --- | --- | --- | --- | --- | --- | --- |
| Strict direct-HR-only pooled | 2y early vs 2y OS | 4 | 0.153 (-2.4 to 2.706) | 0.821 | 0.032 | Both the early-endpoint and OS HR were directly reported. |
| Strict direct-HR-only pooled | 2y early vs 3y OS | 3 | 0.856 (-1.102 to 2.813) | 0.113 | 0.969 | Both the early-endpoint and OS HR were directly reported. |
| Strict direct-HR-only pooled | 3y early vs 5y OS | 4 | 1.136 (-0.59 to 2.862) | 0.105 | 0.8 | Both the early-endpoint and OS HR were directly reported. |
| Strict direct-HR-only pooled | 5y early vs 5y OS | 4 | 1.136 (-0.59 to 2.862) | 0.105 | 0.8 | Both the early-endpoint and OS HR were directly reported. |
| Eligibility-expanded direct-HR-only pooled | 2y early vs 2y OS | 5 | 0.31 (-1.285 to 1.905) | 0.58 | 0.113 | Both the early-endpoint and OS HR were directly reported. |
| Eligibility-expanded direct-HR-only pooled | 2y early vs 3y OS | 3 | 0.856 (-1.102 to 2.813) | 0.113 | 0.969 | Both the early-endpoint and OS HR were directly reported. |
| Eligibility-expanded direct-HR-only pooled | 3y early vs 5y OS | 7 | 0.846 (0.019 to 1.673) | 0.047 | 0.581 | Both the early-endpoint and OS HR were directly reported. |
| Eligibility-expanded direct-HR-only pooled | 5y early vs 5y OS | 7 | 0.846 (0.019 to 1.673) | 0.047 | 0.581 | Both the early-endpoint and OS HR were directly reported. |

The direct-HR-only models exclude every pair containing a Kaplan–Meier reconstructed HR. Endpoint-specific direct pairs were generally too sparse, so DFS and RFS were pooled only for this explicitly labeled source-restriction sensitivity. EFS and broad-taxonomy evidence were excluded.

**Part E. Co-primary conditional errors-in-variables analysis over assumed within-trial correlation**

| **Endpoint** | **Comparison** | **Trials, n** | **Assumed ρ** | **Slope** | **Profile 95% CI** | **Trial-bootstrap 95% CI** | **LR P value** |
| --- | --- | --- | --- | --- | --- | --- | --- |
| DFS | 2y early vs 2y OS | 12 | 0 | 0.011 | -0.502 to 0.521 | -0.198 to 0.177 | 0.966 |
| DFS | 2y early vs 2y OS | 12 | 0.25 | 0.104 | -0.42 to 0.582 | -0.029 to 0.31 | 0.674 |
| DFS | 2y early vs 2y OS | 12 | 0.5 | 0.191 | -0.322 to 0.614 | 0.056 to 0.445 | 0.424 |
| DFS | 2y early vs 2y OS | 12 | 0.75 | 0.264 | -0.215 to 0.611 | 0.118 to 0.614 | 0.24 |
| DFS | 2y early vs 3y OS | 4 | 0 | 0.41 | -0.33 to 1.356 | 0.056 to 0.464 | 0.254 |
| DFS | 2y early vs 3y OS | 4 | 0.25 | 0.503 | -0.254 to 1.353 | 0.228 to 0.63 | 0.165 |
| DFS | 2y early vs 3y OS | 4 | 0.5 | 0.599 | -0.127 to 1.306 | 0.387 to 0.842 | 0.091 |
| DFS | 2y early vs 3y OS | 4 | 0.75 | 0.689 | 0.061 to 1.18 | 0.536 to 1.057 | 0.037 |
| DFS | 2y early vs 5y OS | 3 | 0 | 0.485 | -0.351 to 1.589 | 0.199 to 0.542 | 0.205 |
| DFS | 2y early vs 5y OS | 3 | 0.25 | 0.568 | -0.249 to 1.545 | 0.347 to 0.615 | 0.141 |
| DFS | 2y early vs 5y OS | 3 | 0.5 | 0.654 | -0.124 to 1.458 | 0.497 to 0.735 | 0.084 |
| DFS | 2y early vs 5y OS | 3 | 0.75 | 0.74 | Bound not estimable | 0.639 to 0.93 | 0.038 |
| DFS | 3y early vs 3y OS | 4 | 0 | 0.414 | -0.357 to 1.401 | 0.058 to 0.475 | 0.266 |
| DFS | 3y early vs 3y OS | 4 | 0.25 | 0.511 | -0.277 to 1.4 | 0.22 to 0.676 | 0.173 |
| DFS | 3y early vs 3y OS | 4 | 0.5 | 0.611 | -0.146 to 1.352 | 0.385 to 0.905 | 0.095 |
| DFS | 3y early vs 3y OS | 4 | 0.75 | 0.705 | 0.054 to 1.218 | 0.514 to 1.144 | 0.039 |
| DFS | 3y early vs 5y OS | 3 | 0 | 0.495 | -0.317 to 1.651 | 0.198 to 0.554 | 0.21 |
| DFS | 3y early vs 5y OS | 3 | 0.25 | 0.581 | -0.257 to 1.607 | 0.353 to 0.627 | 0.146 |
| DFS | 3y early vs 5y OS | 3 | 0.5 | 0.67 | -0.141 to 1.516 | 0.499 to 0.802 | 0.088 |
| DFS | 3y early vs 5y OS | 3 | 0.75 | 0.756 | 0.05 to 1.343 | 0.632 to 1.02 | 0.041 |
| DFS | 5y early vs 5y OS | 3 | 0 | 0.457 | -0.233 to 1.419 | 0.198 to 0.488 | 0.182 |
| DFS | 5y early vs 5y OS | 3 | 0.25 | 0.526 | Bound not estimable | 0.353 to 0.55 | 0.127 |
| DFS | 5y early vs 5y OS | 3 | 0.5 | 0.597 | -0.091 to 1.287 | 0.499 to 0.707 | 0.078 |
| DFS | 5y early vs 5y OS | 3 | 0.75 | 0.667 | 0.058 to 1.159 | 0.632 to 0.868 | 0.038 |
| RFS | 2y early vs 2y OS | 7 | 0 | 0.758 | -0.337 to 2.169 | -0.253 to 1.324 | 0.169 |
| RFS | 2y early vs 2y OS | 7 | 0.25 | 0.781 | -0.36 to 2.106 | -0.645 to 1.442 | 0.166 |
| RFS | 2y early vs 2y OS | 7 | 0.5 | 0.824 | -0.379 to 2.067 | -0.694 to 1.516 | 0.158 |
| RFS | 2y early vs 2y OS | 7 | 0.75 | 0.885 | -0.388 to 2.028 | -0.656 to 1.711 | 0.144 |
| RFS | 2y early vs 3y OS | 4 | 0 | 0.716 | -0.033 to 1.847 | 0.228 to 0.867 | 0.076 |
| RFS | 2y early vs 3y OS | 4 | 0.25 | 0.813 | 0.02 to 1.82 | 0.44 to 0.943 | 0.045 |
| RFS | 2y early vs 3y OS | 4 | 0.5 | 0.915 | Bound not estimable | 0.675 to 1.097 | 0.022 |
| RFS | 2y early vs 3y OS | 4 | 0.75 | 1.017 | 0.39 to 1.613 | 0.904 to 1.223 | 0.007 |
| RFS | 3y early vs 3y OS | 4 | 0 | 0.557 | -0.037 to 1.313 | 0.218 to 0.794 | 0.066 |
| RFS | 3y early vs 3y OS | 4 | 0.25 | 0.611 | Bound not estimable | 0.424 to 0.898 | — |
| RFS | 3y early vs 3y OS | 4 | 0.5 | 0.665 | 0.095 to 1.213 | 0.549 to 1.036 | 0.026 |
| RFS | 3y early vs 3y OS | 4 | 0.75 | 0.712 | 0.199 to 1.109 | 0.586 to 1.175 | 0.013 |

Let xᵢ=−log(HR early), yᵢ=−log(HR OS), with standard errors sₓᵢ and sᵧᵢ. For each fixed ρ∈{0, 0.25, 0.50, 0.75}, the custom conditional likelihood assumed yᵢ−α−βxᵢ~N(0,Vᵢ), where Vᵢ=sᵧᵢ²+β²sₓᵢ²−2βρsₓᵢsᵧᵢ+τ². The custom base-R routine jointly estimated α, β, and log(τ) by maximum likelihood with optim(method='L-BFGS-B'). Profile 95% CIs fixed β, reoptimized α and log(τ), and used uniroot to locate NLLmin+χ²(1,0.95)/2. LR P values tested β=0. If profiling failed or no cutoff crossing was found within the β search range (−20 to 20), that bound was reported as not estimable and was not imputed. Trial-bootstrap intervals used 1,000 resamples. All measurement-error models with n=3–4 are exploratory; their assumption-specific LR P values are unadjusted for multiplicity and are not evidence of surrogacy.

**Part F. EFS and PFS/PFS2 supplementary analyses**

| **EFS comparison** | **Trials, n** | **Trials** | **Analysis status** |
| --- | --- | --- | --- |
| 2y early vs 2y OS | 2 | CREST; DIPPER | Descriptive only; no EFS regression |
| 2y early vs 3y OS | 2 | CREST; DIPPER | Descriptive only; no EFS regression |
| 2y early vs 5y OS | 0 | None | Descriptive only; no EFS regression |
| 3y early vs 3y OS | 2 | CREST; DIPPER | Descriptive only; no EFS regression |
| 3y early vs 5y OS | 0 | None | Descriptive only; no EFS regression |
| 5y early vs 5y OS | 0 | None | Descriptive only; no EFS regression |

| **PFS analysis** | **Trials, n** | **Slope (95% CI)** | **P value** | **Weighted R²** | **Population/role** |
| --- | --- | --- | --- | --- | --- |
| PFS only; PFS2 excluded | 4 | 0.575 (-0.29 to 1.44) | 0.103 | 0.804 | Strict plus eligibility-sensitivity; broad taxonomy excluded |
| Legacy combined PFS/PFS2 | 5 | 0.639 (0.145 to 1.134) | 0.026 | 0.85 | Strict plus eligibility-sensitivity; broad taxonomy excluded |

EFS had no strict-primary pair and only two eligibility-sensitivity trials, so it is descriptive. The PFS-only model excludes CheckMate 274 PFS2. Both PFS analyses are supplementary because only one strict-primary conventional PFS pair was available; no broad-taxonomy study is included.
