# Supplementary material for: Linking early and late endpoints in adjuvant immunotherapy trials: an evaluation of multiple clinical outcomes
Source: Oncologist. 2026 Aug 22;31(10):oyag342. doi: 10.1093/oncolo/oyag342 (PMC13584216; doi:10.1093/oncolo/oyag342)
Supplement: Supplementary file 1 [file oyag342_supplementary_data.zip › Figure S1.pdf]

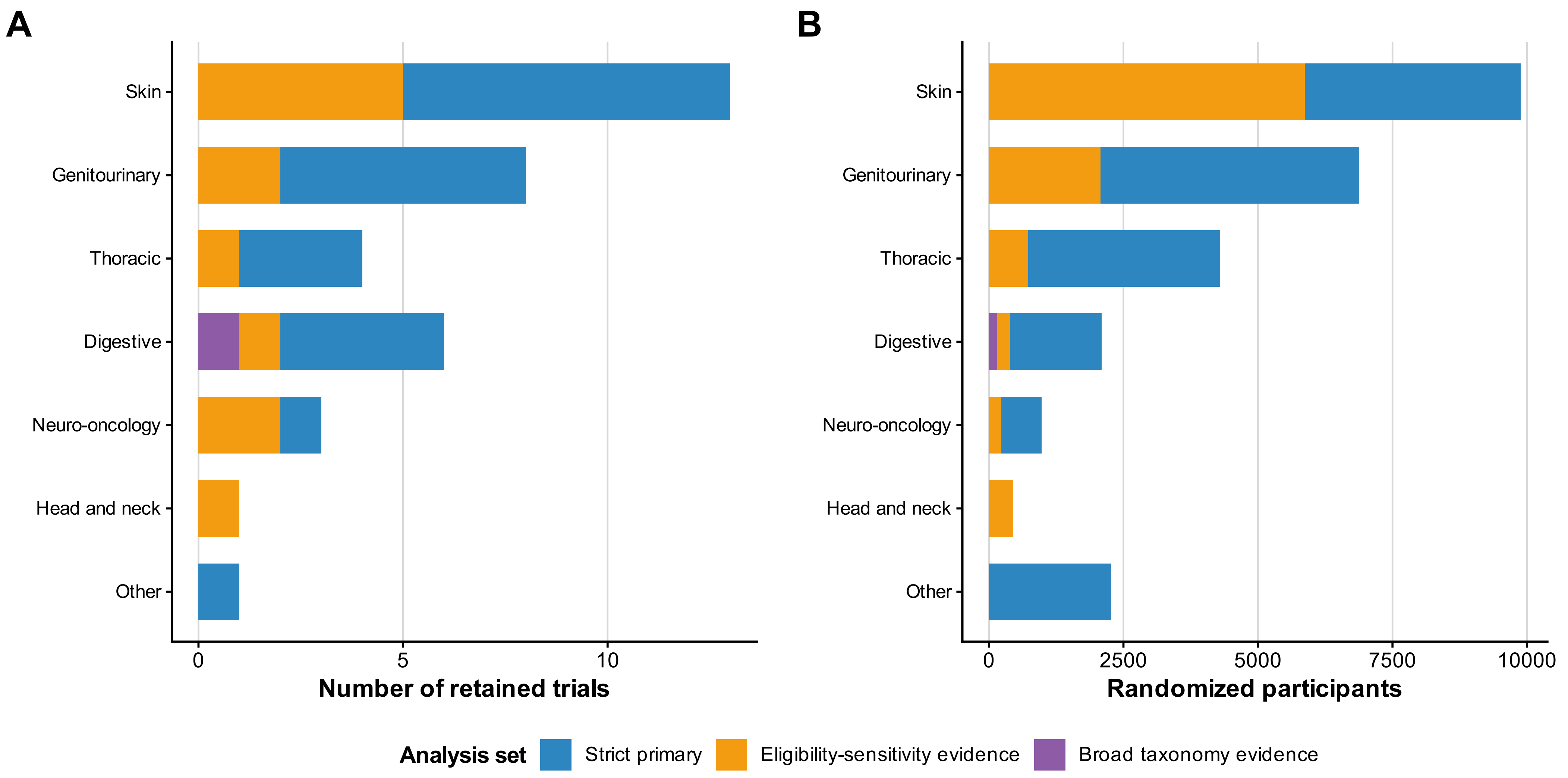
A
B
Skin
Genitourinary
Thoracic
Digestive
Neuro-oncology
Head and neck
Other
0
5
10
Number of retained trials
0
2500
5000
7500
10000
Randomized participants
Analysis set
Strict primary
Eligibility-sensitivity evidence
Broad taxonomy evidence
