# Supplementary material for: Linking early and late endpoints in adjuvant immunotherapy trials: an evaluation of multiple clinical outcomes
Source: Oncologist. 2026 Aug 22;31(10):oyag342. doi: 10.1093/oncolo/oyag342 (PMC13584216; doi:10.1093/oncolo/oyag342)
Supplement: Supplementary file 1 [file oyag342_supplementary_data.zip › Figure S2.pdf]

	Early endpoint	OS status	Paired analysis	QoL contrast	Randomized N
Adjuvant durvalumab ESCC	DFS	Available	Primary	No	86
ADMEC-O	DFS	None	No pair	No	179
AMBASSADOR	DFS	Interim	Primary	No	702
C-POST	DFS	Limited	Primary	Yes	415
CANOPY-A	DFS	Available	Primary	No	1382
CheckMate 274	DFS	Interim	Primary	Yes	709
CheckMate 914 Part A	DFS	None	No pair	No	816
IMmotion010	DFS	Limited	Primary	No	778
IMpower010	DFS	Interim	Primary	No	1005
IMvigor010	DFS	Interim	Primary	No	809
KEYNOTE-564	DFS	Interim	Primary	Yes	994
MAGRIT	DFS	Available	Primary	No	2272
PEARLS/KEYNOTE-091	DFS	Limited	Primary	No	1177
ATTRACTION-5	RFS	Available	Primary	No	755
CheckMate 76K	RFS	None	No pair	Yes	790
EORTC 1325/KEYNOTE-054	RFS	None	No pair	Yes	1019
EORTC 18071	RFS	Available	Primary	Yes	951
IMbrave050	RFS	Limited	Primary	No	668
IMMUNED	RFS	Available	Primary	No	167
MIND-DC	RFS	Available	Primary	No	148
Seviprotimut-L	RFS	Limited	Primary	No	347
Sintilimab HCC	RFS	Limited	Primary	No	198
ACT IV	PFS	Available	PFS only	No	745
